# Mutational topography reflects clinical neuroblastoma heterogeneity

**DOI:** 10.1016/j.xgen.2023.100402

**Published:** 2023-09-07

**Authors:** Elias Rodriguez-Fos, Mercè Planas-Fèlix, Martin Burkert, Montserrat Puiggròs, Joern Toedling, Nina Thiessen, Eric Blanc, Annabell Szymansky, Falk Hertwig, Naveed Ishaque, Dieter Beule, David Torrents, Angelika Eggert, Richard P. Koche, Roland F. Schwarz, Kerstin Haase, Johannes H. Schulte, Anton G. Henssen

**Affiliations:** 1 Experimental and Clinical Research Center (ECRC) of the MDC and Charité Berlin, Berlin, Germany; 2 Department of Pediatric Oncology and Hematology, Charité – Universitätsmedizin Berlin, Corporate Member of Freie Universität Berlin, Humboldt-Universität zu Berlin, Berlin, Germany; 3 Berlin Institute for Medical Systems Biology (BIMSB), Max Delbrück Center for Molecular Medicine in the Helmholtz Association (MDC), Berlin, Germany; 4 Barcelona Supercomputing Center, Joint Barcelona Supercomputing Center – Center for Genomic Regulation – Institute for Research in Biomedicine Research Program in Computational Biology, Barcelona, Spain; 5 Institució Catalana de Recerca i Estudis Avançats (ICREA), Barcelona, Spain; 6 Berlin Institute of Health at Charité – Universitätsmedizin Berlin, Digital Health Center, Berlin, Germany; 7 Center for Epigenetics Research, Memorial Sloan Kettering Cancer Center, New York, NY, USA; 8 Center for Integrated Oncology (CIO), Cancer Research Center Cologne Essen (CCCE), Faculty of Medicine and University Hospital Cologne, University of Cologne, Cologne, Germany; 9 BIFOLD – Berlin Institute for the Foundations of Learning and Data, Berlin, Germany; 10 German Cancer Consortium (DKTK), Partner Site Berlin, and German Cancer Research Center (DKFZ), Heidelberg, Germany

**Keywords:** neuroblastoma, mutational signatures, cancer genomics, tumor evolution, ecDNA, complex rearrangements, structural variation, clinical heterogeneity, mutational processes

## Abstract

Neuroblastoma is a pediatric solid tumor characterized by strong clinical heterogeneity. Although clinical risk-defining genomic alterations exist in neuroblastomas, the mutational processes involved in their generation remain largely unclear. By examining the topography and mutational signatures derived from all variant classes, we identified co-occurring mutational footprints, which we termed mutational scenarios. We demonstrate that clinical neuroblastoma heterogeneity is associated with differences in the mutational processes driving these scenarios, linking risk-defining pathognomonic variants to distinct molecular processes. Whereas high-risk *MYCN*-amplified neuroblastomas were characterized by signs of replication slippage and stress, homologous recombination-associated signatures defined high-risk non-*MYCN*-amplified patients. Non-high-risk neuroblastomas were marked by footprints of chromosome mis-segregation and TOP1 mutational activity. Furthermore, analysis of subclonal mutations uncovered differential activity of these processes through neuroblastoma evolution. Thus, clinical heterogeneity of neuroblastoma patients can be linked to differences in the mutational processes that are active in their tumors.

## Highlights

• Clinical neuroblastoma heterogeneity is linked to differences in mutational processes
• HR *MYCN*-amp neuroblastomas are characterized by signs of replication slippage and stress
• HR non-*MYCN*-amp neuroblastomas are defined by HRD signatures
• Non-HR neuroblastomas are marked by chromosome mis-segregation and TOP1 activity

## Graphical abstract

**Figure:**
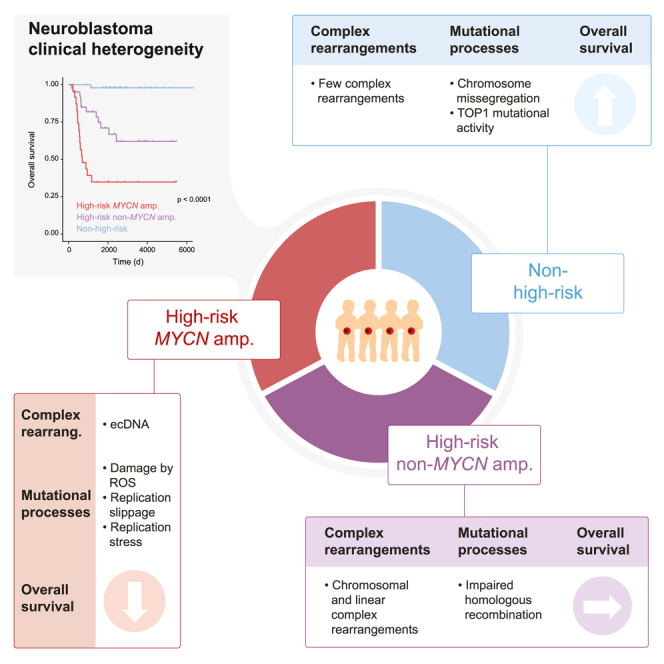

## Teaser

Rodriguez-Fos et al. reveal that the strong clinical heterogeneity observed in neuroblastoma patients is associated with differences in the activity of specific mutational processes. Their work suggests that replication slippage/stress, homologous recombination deficiency, and chromosome mis-segregation have important implications in neuroblastoma evolution and survival.

## Introduction

The presence of somatic mutations is a hallmark of cancer genomes.^1^ Diverse types of somatic mutations are a result of different endogenous and/or exogenous mutational processes, including replication errors, exposure to DNA-damaging agents, expression of developmentally restricted recombinases,^2^ and errors in DNA-repair mechanisms.^3^ These processes imprint characteristic mutational patterns in the genome, termed mutational signatures.^4^ Recent analyses in multiple cancer types have extracted signatures associated with single-nucleotide variants (SNVs),^5^^,^^6^ small insertions and deletions (indels),^6^ copy-number alterations (CNAs),^7^^,^^8^ and structural variants (SVs).^9^ Some signatures are linked to known biological processes active in cancer, whereas others have yet-unknown etiologies.^5^^,^^6^^,^^10^ In contrast with mutational signatures derived from SNVs and indels, those derived from CNAs and SVs remain difficult to classify, and their etiologies remain largely unknown. Various complex SV classes have recently been defined on the basis of their topography, e.g., circular extrachromosomal DNA^11^ (ecDNA), chromothripsis,^12^ chromoplexy,^13^ templated insertion chains (TICs),^14^ breakage-fusion-bridge cycles^15^ (BFBs), complex non-cyclic amplicons^16^ (CnCs), tyfona, rigma, and pyrgo,^17^ among others. Even though the study of mutational signatures and structural variant patterns has advanced in recent years, it remains largely unclear how different mutational processes and variant topographies are mechanistically linked.

Neuroblastoma is a pediatric solid tumor characterized by strong clinical heterogeneity.^18^^,^^19^^,^^20^ Patients can be classified into different disease risk groups depending on their clinical, genetic, and molecular characteristics, namely low-, intermediate- or high-risk groups, with the latter presenting poor prognosis despite intensive therapy and surgery.^21^ Cytogenetic and genomic studies have identified many neuroblastoma risk-group-specific alterations^19^^,^^22^^,^^23^^,^^24^^,^^25^^,^^26^ such as ecDNA and segmental chromosomal gains/losses in high-risk patients or whole chromosomal aberrations in low-risk patients.^27^^,^^28^^,^^29^ Complex rearrangements involving oncogene amplicons are particularly frequent in neuroblastoma.^30^^,^^31^ Recent studies have linked ecDNA to other complex variants such as chromothripsis and BFBs^32^^,^^33^ and have identified circular recombination as a potential means of ecDNA evolution in neuroblastomas.^33^ What causes these pathognomonic alterations remains largely unknown. We hypothesized that co-occurrence analyses of mutational signatures derived from independent variant types may identify new principles of neuroblastoma mutagenesis that explain the differences in variant patterns observed across clinical risk groups.

## Results

### SNV-based mutational signatures differ between neuroblastoma risk groups

**Figure 1:**
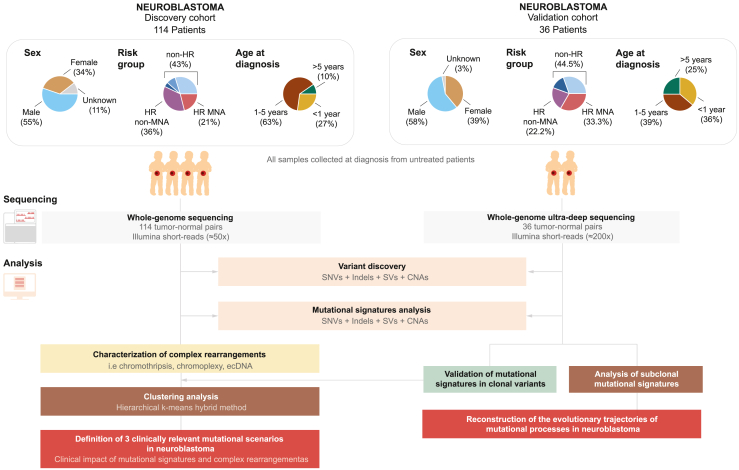
Cohort characteristics and analysis strategy Top: description of the distribution (in percentage) of the 150 neuroblastoma samples from the discovery (n = 114; see also Figure S1) and validation (n = 36) cohorts, within sex groups (female, male, and unknown), risk groups (HR MNA, HR non-MNA, and non-HR: low-risk; low-risk stage 4S; intermediate-risk), and age at diagnosis groups (<1 year old, 1–5 years old, and >5 years old). Middle: summary of the sequencing datasets available for our analysis. WGS matched tumor-normal pairs. Bottom: description of the main steps carried out in our study, starting with the variant discovery and the mutational signatures analysis, followed by validation of the signatures and subclonal SNV-based signatures extraction, characterization of the complex rearrangements present in our samples, unsupervised clustering, and definition of the three clinically relevant neuroblastoma mutational scenarios presented in this work.

To explore the link between different mutational patterns from independent variant types in neuroblastoma, we analyzed two publicly available, previously published cohorts of whole genomes from 103 matched tumor-normal pairs as well as 11 unpublished whole genomes, which were all derived from neuroblastoma patients treated according to the same clinical protocol. All sex, age, and disease stages were represented in this dataset (Figures 1 and S1). Pathognomonic chromosomal and driver gene alterations were identified at a comparable frequency in our cohort as described in other neuroblastoma cohorts^26^^,^^30^^,^^34^ (Figure S1A). Recurrently mutated genes were comparable to those found in other cohorts (Figure S1A), evidencing that this cohort was representative for clinically heterogeneous neuroblastomas.

**Figure 2:**
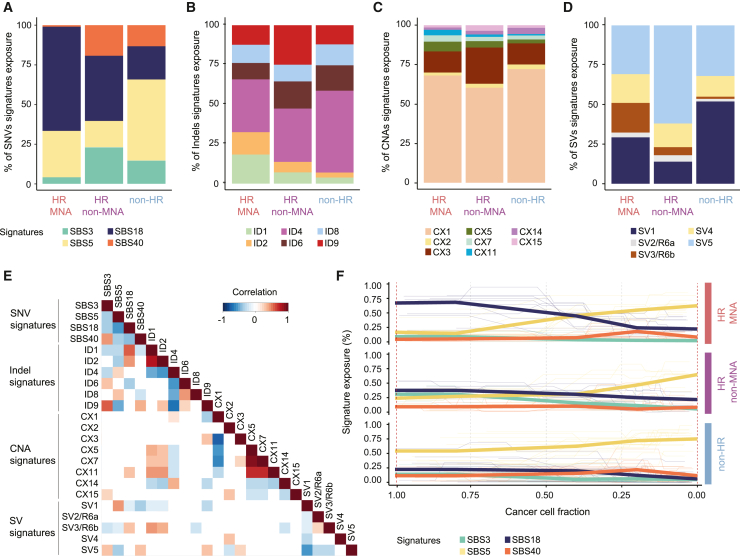
Distribution and correlation of SNV-, indel-, SV-, and CNA-associated signatures in neuroblastoma clinical risk groups (A) Exposure (in percentage) of the four SNV-associated signatures (SBSs) identified in our neuroblastoma discovery cohort by clinical risk group (n = 114). Each color displays a different signature: SBS3, SBS5, SBS18, and SBS40 (see also Figures S2A–S2C). Columns are ordered by neuroblastoma clinical risk classification. (B) Exposure (in percentage) of the six indel signatures (ID; insertions and deletions <50 bp) identified in our cohort by clinical risk group. Each color displays a different signature: ID1, ID2, ID4, ID6, ID8, and ID9 (see also Figures S2D–S2F). Columns are ordered by neuroblastoma clinical risk classification. (C) Exposure (in percentage) of the eight CNA signatures (CX; gains, losses, amplifications, and homozygous deletions) extracted in our cohort by clinical risk group. Each color displays a different signature: CX1, CX2, CX3, CX5, CX7, CX11, CX14, and CX15 (see also Figures S2G and S2H). Columns are ordered by neuroblastoma clinical risk classification. (D) Exposure (in percentage) of the six SV signatures (SV; deletions, duplications, translocations, and inversions) identified in our cohort by clinical risk group. Each color displays a different signature: SV1–SV6. SV2, and SV3 correspond to reference signatures R6b and R6a, respectively (see also Figures S3A–S3D). Columns are ordered by neuroblastoma clinical risk classification. (E) Heatmap depicting the positive (red) and negative (blue) correlations between the signatures associated with different types of mutations (SNV, indel, SV, and CNA). Colors display the Spearman correlation coefficient. Only significant correlations are included (p < 0.05, false discovery rate [FDR] correction). (F) Activity trajectories of the four SNV-associated signatures (SBSs) per cancer cell fraction by clinical risk group in the validation cohort (n = 36). Thick lines correspond to average exposure for all samples. Thin lines correspond to per-sample exposure (see also Figure S4).

To provide a comprehensive overview of the mutational processes involved in neuroblastoma, we first analyzed the SNV trinucleotide context (Figures 2A, 2E, and S2A–S2C; Tables S1, S2, and S3). We recurrently identified four SNV-based signatures^6^ (SBSs) in this cohort, indicating that associated mutational processes are active in neuroblastoma. In line with previous reports,^35^ SBS3 and SBS18, signatures experimentally linked to defective homologous recombination repair (HRR) and reactive oxygen species (ROS),^36^^,^^37^ respectively, were recurrently identified in this cohort (Figures 2A, S2A, and S2B). Whereas SBS18 was predominantly observed in high-risk *MYCN*-amplified patients (p < 1.9 × 10^−4^), SBS3 was observed to a significantly lower degree in this risk group (p < 5.4 × 10^−5^), displaying a negative correlation with signature SBS18, and was more prevalent in high-risk non-*MYCN*-amplified patients. Similarly, SBS40 and SBS5, clock-like signatures associated with patient age,^10^^,^^38^ were highly anti-correlated with SBS18 in this cohort. Clock-like signature SBS5 was inversely associated with clinical risk and most prevalent in non-high-risk patients (p < 3.3 × 10^−4^). Thus, SNV-based mutational signatures attributed to defective HRR and ROS are prevalent and differentially active across neuroblastoma risk groups and serve as the basis for co-occurrence analyses to link mutational etiologies across variant types.

### Mutational processes change during high-risk neuroblastoma evolution

We and others recently described that some complex rearrangements occur at different times throughout neuroblastoma development.^39^^,^^40^ To link mutational processes driving SNVs to such complex rearrangement types as well as other variant types, we determined the timing of mutational processes during neuroblastoma evolution. To do so, we performed ultra-deep sequencing at an average coverage of 200× in an independent cohort of 36 neuroblastomas also derived from patients at the time of diagnosis (Figure 1). We validated 69.6% of the signatures extracted in the discovery cohort (SNV-, indel-, CNA-, and SV-based; n = 114). The frequency of the mutational signatures across risk groups was significantly linearly correlated between both cohorts (r^2^ = 0.61, p = 4.4 × 10^−5^; Figures S3E and S3F; Tables S2 and S3). Thus, the signatures identified in this study are robustly and reproducibly detectable in independent neuroblastoma cohorts. Next, we reconstructed the evolutionary trajectories of mutational processes based on the density of mutation frequencies and changes in mutational signature activities (Figures 2F and S5; Table S4). Through the reconstruction of changes in signature activity across different cancer cell fractions (CCFs), this approach enables the inference of mutational evolution in tumors.^41^ Signatures SBS18 and SBS3 were predominantly found at higher CCFs in high-risk patients (p < 3.14 × 10^−2^ and p < 2.7 × 10^−4^, respectively), whereas SBS5 activity was dominant in their subclones. This indicates that mutations resulting from ROS or HRR deficiency are more frequent in early high-risk neuroblastoma development, while cell-intrinsic mutational processes accumulate over time and occur later in neuroblastoma evolution. Non-high-risk patients, on the other hand, did not show any changes in mutational trajectories, exhibiting a predominance of SBS5-associated mutations across CCFs (p < 4.8 × 10^−3^), i.e., a stable frequency of mutagenic processes throughout tumor evolution. This indicates that mutational processes involved in neuroblastoma initiation and progression are distinct and differ between risk groups. While non-high-risk neuroblastomas are characterized by continuous exposure to cell-intrinsic mutational processes, mutational processes in high-risk neuroblastomas seem to switch between early and late stages of tumor evolution.

### Indel-based signatures confirm differences in mutational processes active in neuroblastoma risk groups

Co-occurrence analyses of mutational signatures from independent variant types that are experimentally linked to molecular etiologies such as SNVs and indels are crucial to determining the mutational processes active in a tumor.^3^^,^^5^^,^^6^ Based on the insertion and deletion lengths and genomic context, including repetitive sequences and microhomology, we identified six indel-based signatures^6^ (IDs) in neuroblastoma genomes (Figures 2B, 2E, and S2D–S2F; Tables S1 and S3). ID1 and ID2, both characterized by 1 bp insertions and deletions at long thymine homopolymers attributed to replication slippage and found in most cancer entities,^6^ were also recurrently identified in neuroblastoma genomes. These signatures were significantly more frequent in patients with higher clinical risk, particularly in high-risk *MYCN*-amplified patients (p < 2 × 10^−4^). ID1 and ID2 correlated positively with SNV signature SBS18, which indicates co-occurrence of DNA damage by ROS and replication slippage in *MYCN*-amplified neuroblastomas. ID6, characterized by larger deletions (>5 bp) with larger microhomology, was also recurrently observed in our cohort. In agreement with their common etiology associated with defective HRR, ID6 followed the same prevalence distribution as SBS3, corroborating the predominance of defective HRR in high-risk non-*MYCN*-amplified neuroblastomas (p < 1.8 × 10^−2^). ID4, a signature enriched for deletions (>1 bp) at repeats and microhomology, recently associated experimentally with TOP1 mutational activity in cancer and healthy cells,^42^ on the other hand was significantly more prevalent in non-high-risk patients (p < 1.68 × 10^−3^). ID4 displayed an association with SBS5 and high anti-correlation with all the other indel signatures, notably with the ones related to SBS3 (ID6, ID8, ID9) or SBS18 (ID1, ID2). This is in line with previous works in other cancer entities describing ID4 as mutually exclusive with signatures ID1 and ID2^6^ and points to functional impairment of topoisomerase 1 as a source of mutagenesis in non-high-risk neuroblastoma. The co-occurrence of SNV-based and indel-based signatures associated with the same mutational processes further supports that distinct mutational processes contribute to mutagenesis in different neuroblastoma risk groups.

### High-risk *MYCN*-amplified neuroblastoma genomes are defined by replication-stress-related CNA patterns

Whereas the mutational processes involved in SNV and indel generation have been extensively explored, less is known about the origin of CNAs and SVs. Recent reports suggest that CNA patterns can be grouped into signatures, offering more insights into molecular processes involved in their generation.^7^^,^^8^ As neuroblastomas from different risk groups are known to harbor different CNA patterns,^19^^,^^22^^,^^23^^,^^26^^,^^27^^,^^28^^,^^29^ we reasoned that co-occurrence analysis of CNA signatures with SNV and indel signatures may uncover processes linked to CNA genesis in neuroblastoma. Thus, we evaluated the presence of recently established CNA-associated signatures^8^ (CXs) in our cohort (Figures 2C, 2E, S2G, and S2H; Tables S1 and S3). CX1 was the most active CNA signature found in this cohort (Figure 2C). CX1 together with CX14, both associated with chromosomal arm changes potentially caused by chromosome mis-segregation, were most prevalent in non-high-risk patients (p < 9.1 × 10^−4^), in line with previous works associating whole-chromosome alterations with lower clinical risk.^43^ CX3, linked to defective HRR, was most frequent in high-risk non-*MYCN*-amplified patients (p < 2.1 × 10^−2^), further corroborating the role of deficient HRR as a risk-group-defining mutational process in neuroblastoma. CX11, a signature attributed to replication-stress-mediated focal amplifications, and SBS18 significantly co-occurred in high-risk neuroblastomas, raising the possibility that ROS-induced replication stress may contribute to the generation of focal amplifications in these tumors. CX5, CX7, and CX11 were most frequent in high-risk *MYCN*-amplified patients (p < 4.4 × 10^−3^). Notably, CX5 and CX11, both attributed to replication stress, were significantly associated with the presence of indel-based signatures ID1 and ID2, which result from replication slippage. This further highlights replication-stress-associated mutational processes as contributors to mutagenesis in high-risk neuroblastomas. Thus, co-occurrence analyses of CNA, SNV, and indel signatures revealed that whereas focal amplifications typically found in *MYCN*-driven neuroblastomas are linked to signs of replication stress and slippage, CNAs in high-risk non-*MYCN*-amplified neuroblastomas are associated with defective HRR, and chromosome mis-segregation patterns are a general feature of all neuroblastomas across risk groups.

### *De novo* structural variant signature analysis identifies neuroblastoma-specific patterns

**Figure 3:**
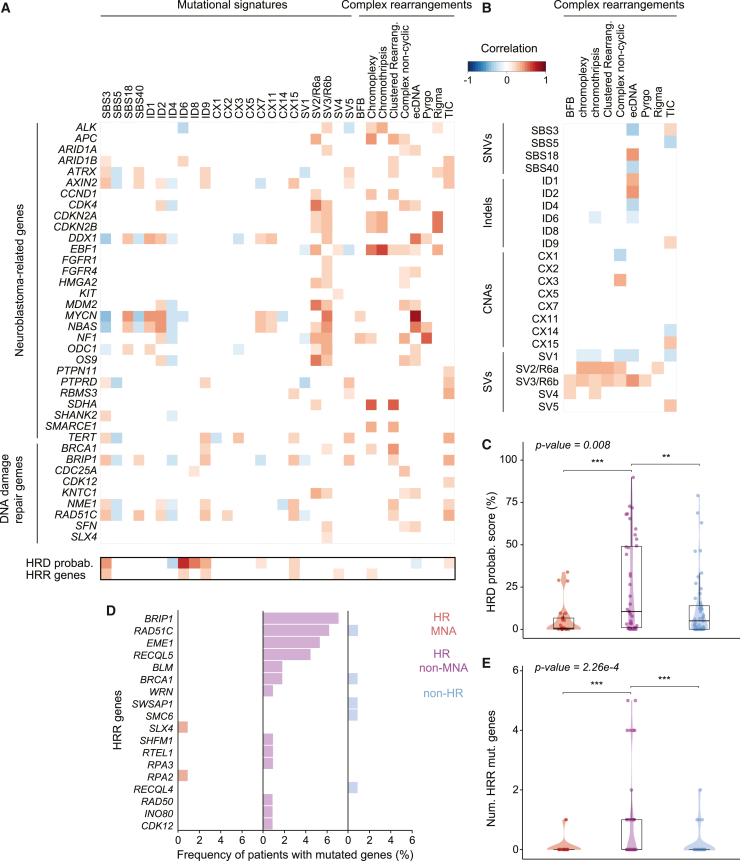
Co-occurrence of mutational signatures, complex rearrangements, and cancer-related gene alterations in neuroblastoma (A) Heatmap showing the correlations between mutated neuroblastoma driver genes and DNA-damage-repair genes and all the mutational signatures and complex rearrangement types identified in our cohort (n = 114). Below are rows showing the correlation between HRD probability score, mutations in HRR genes, and the mutational signature exposures and complex rearrangements identified in our cohort. In both heatmaps, colors display the Spearman correlation coefficient. Only significant correlations are included (p < 0.05, FDR correction). (B) Heatmap depicting the correlations between the signatures associated with different variant types (SNV, indel, SV, and CNA) and the nine complex rearrangement classes identified in our cohort (n = 114). (C) Box plot comparing the distribution of HRD probability scores across neuroblastoma risk groups (HR MNA, HR non-MNA, and non-HR). Each dot represents a patient. To assess whether there are differences between risk groups, we used the non-parametric Kruskal-Wallis test (p value in the upper-left corner). (D) Frequency of patients with mutated HRR genes across the three neuroblastoma risk groups. (E) Box plot comparing the distribution of HRR mutated genes across neuroblastoma risk groups (HR MNA, HR non-MNA, and non-HR). Each dot represents a patient. To assess whether there are differences between risk groups, we used the non-parametric Kruskal-Wallis test (p value in the upper-left corner). Pairwise comparisons were done using the non-parametric Wilcoxon rank-sum test. Significance: ∗p < 0.1, ∗∗p < 0.05, ∗∗∗p < 0.01. All analyses were performed in the discovery cohort (n = 114).

We and others have shown that complex SVs are prevalent in neuroblastomas.^30^^,^^33^ The mechanisms involved in their generation, however, are currently largely unknown. Co-occurrence analyses with mutational signatures from other variant types facilitate the investigation of etiologies associated with distinct SV patterns. To this end, we performed *de novo* discovery of SV signatures^9^ (SVs) based on the type, size, and clustering of SVs (Figures 2D, 2E, and S3A–S3D; Tables S1, S2, and S3). Comparing the SV signatures we extracted to SV signatures identified in other tumor entities,^9^ we determined that three signatures were specific to this cohort (SV1, SV4, and SV5), whereas two SV-based signatures (SV2 and SV3, corresponding to R6a and R6b) had already been identified in other cancer types. SV1, defined by simple deletions smaller than 1 kb, was predominantly detected in non-high-risk patients (p < 3.3 × 10^−2^) and showed a negative correlation with all the other SV-based signatures. It was positively correlated with SBS5, a clock-like signature, suggesting it may have similar origins. SV2/R6a and SV3/R6b, characterized by clustered intrachromosomal rearrangements around 1–10 Mb and larger than 10 Mb, respectively, were linked to the presence of focal oncogene amplifications (Figures 3A and 3B). This is in line with results from previous reports in other tumor entities^9^ and indicates that these signatures could represent footprints of the molecular processes involved in the generation of high-level oncogene amplification. Consequently, these signatures were highly prevalent in high-risk *MYCN*-amplified neuroblastomas (p < 2.5 × 10^−2^), which contain focal amplifications. SV3/R6b was also correlated with the exposure of replication slippage signature (Figure 2E), suggesting a role of this mutational process in the generation of complex amplicons in neuroblastoma, as previously proposed in other tumor entities.^44^^,^^45^^,^^46^ Notably, the overall frequency of clustered SV-based signatures (SV2/R6a, SV3/R6b, and SV4) was correlated with clinical risk. Thus, distinct complex SV patterns prevalent in high-risk neuroblastomas can be categorized using SV-based mutational signatures and significantly co-occur with signatures based on other variant types, raising the possibility that similar mutagenic processes contribute to their generation.

### Complex structural rearrangement topography is linked to distinct mutational processes across neuroblastoma risk groups

**Figure 4:**
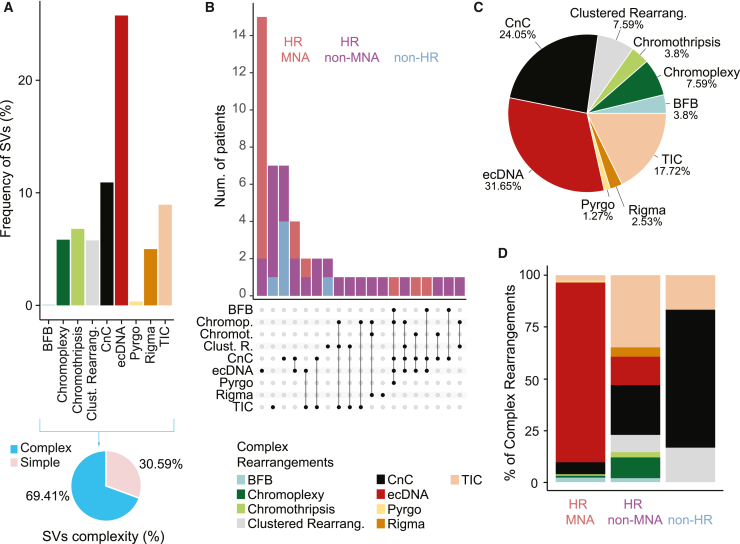
Co-occurrence and distribution of complex rearrangements in neuroblastoma (A) Top: frequency of SVs involved in each complex rearrangement type, in percentage. Bottom: pie chart showing the percentage of SVs involved in complex and simple events in our cohort. (B) Upset plot depicting the co-occurrence of the different types of complex SV patterns within patients. The number of patients with each combination of rearrangements is shown in the top histogram (colors display the risk group for each patient). (C) Pie chart showing the frequency in percentage of each complex rearrangement type in the whole neuroblastoma cohort. (D) Relative frequency in percentage of the nine different complex rearrangement types identified in our cohort by clinical risk group (HR MNA, HR non-MNA, and non-HR). All analyses were performed in the discovery cohort (n = 114). See also Figure S5.

Recent reports have reclassified complex rearrangements topographically, considering both CNA- and SV-associated features.^14^^,^^17^^,^^47^ Even though a subset of these rearrangement patterns has been observed in some neuroblastomas, e.g., ecDNA and chromothripsis,^30^^,^^48^ their prevalence across neuroblastoma risk groups and their co-occurrence with mutational signatures has not yet been determined. To examine the topography of complex rearrangements in neuroblastoma, we used three state-of-the-art complementary algorithms, enabling the identification and reconstruction of nine complex variant classes: (1) chromothripsis; (2) BFBs; (3) ecDNA; (4) CnCs; (5) chromoplexy; (6) TICs; (7) rigma, a cluster of simple deletions; (8) pyrgo, a cluster of tandem duplications (Figure 4); and (9) unclassified regions with high SV density, termed clustered rearrangements. The fraction of SVs assigned to complex variant patterns corresponded to 69.41% and differed between risk groups (p < 2.39 × 10^−9^; Figures 4A and S5D), suggesting that structural variation in high-risk neuroblastomas is predominantly complex. In line with previous observations,^30^ ecDNA was the most frequently observed SV pattern in neuroblastomas, detectable in 31.65% of the patients (Figures 4B and 4C; Table S6). The predominance of ecDNA defined high-risk *MYCN*-amplified patients (Figures 4D, S5A, and S5E–S5N; p < 1.3 × 10^−7^), in line with the fact that *MYCN* amplifications most often occur in the form of ecDNA.^30^^,^^49^ *MYCN* amplifications detected in whole-genome sequencing (WGS) were confirmed cytogenetically using fluorescence *in situ* hybridization (FISH) and classified as either ecDNA or homogenously staining regions (Table S5). A subset of ecDNA-harboring neuroblastomas also contained instances of chromothripsis and/or BFBs, in line with previous reports showing that these processes can contribute to ecDNA generation and evolution.^32^^,^^33^ Most ecDNAs, however, did not co-occur with any other complex rearrangement (Figure 4B), suggesting that they are a result of other processes. Simple DNA circularization due to replication slippage can lead to ecDNA generation,^44^ which would be consistent with our co-occurrence analyses results (Figure 3B). High-risk non-*MYCN*-amplified patients, on the other hand, exhibited a high variety of mostly linear chromosomal complex rearrangements (Figures 4B, 4D, S9A, and S9E–S9N), including unclassified clustered rearrangements. Through manual reconstruction of these unclassified events, we identified two complex SV patterns of local n-jumps^14^ named duplication-inverted-triplication-duplication (Dup-Trp-Dup) and duplications linked by inverted segments (Dup-invDup) (Figure S5O), which had not been previously described in neuroblastoma.

Exploring the potential etiologies of complex SV patterns, we found that signatures characterized by clustered SVs (SV2/R6a, SV3/R6b, and SV4) were correlated with the presence of complex amplicon topography such as chromothripsis, BFBs, and ecDNA (Figure 3B). Furthermore, SV5, characterized by simple deletions and duplications along with non-clustered translocations, was linked to TICs, based on its SV pattern composition and the significant co-occurrence with this complex rearrangement pattern (Figures 3B and S3C). SV5 was correlated with signatures of HRR deficiency, suggesting its role in the generation of TICs.

**Figure 5:**
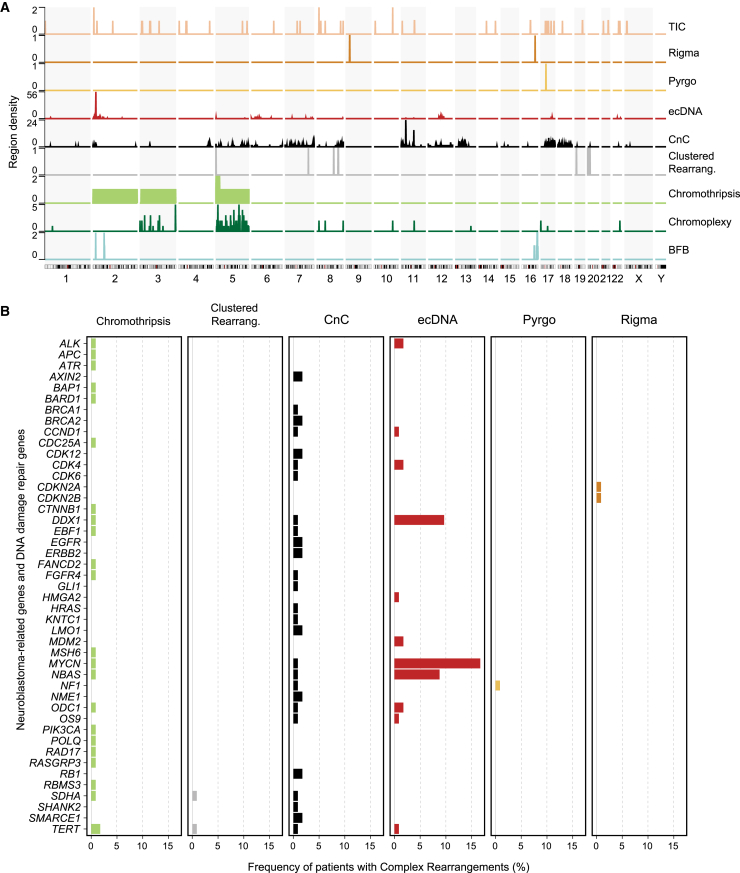
Genomic distribution of topographically defined complex rearrangement patterns in neuroblastoma (A) Density plot showing the number of regions affected by complex rearrangements per chromosome across the whole human genome. Each row/color corresponds to a different type of rearrangement. (B) Frequency of patients with complex rearrangements affecting neuroblastoma driver genes or DNA-damage-repair genes. Each column and color corresponds to a different type of rearrangement. All analyses were performed in the discovery cohort (n = 114). See also Figure S5.

Complex SVs did not occur uniformly throughout the genome (Figures 5 and S5C). Known neuroblastoma-related genes such as *MYCN*, *TERT*, *ODC1, CDK4,* and *MDM2* were recurrently affected by different classes of complex rearrangements including ecDNA, chromothripsis, and CnCs. Thus, topographically distinct complex variant patterns are more common in neuroblastoma than previously anticipated and recurrently occur at sites of cancer-related loci, suggesting their functional oncogenic importance.

### Risk-group-defining mutational scenarios emerge through integration of mutational signatures and complex rearrangement patterns in neuroblastoma

**Figure 6:**
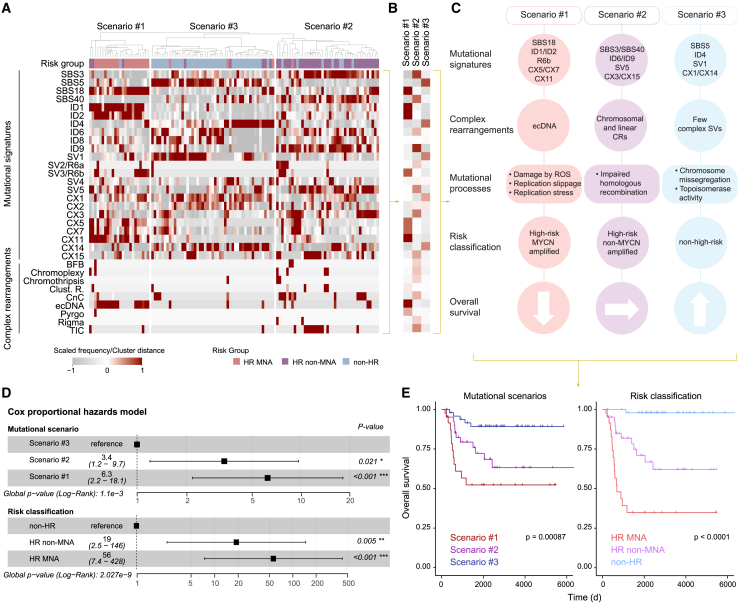
Characterization and definition of mutational scenarios linked to clinical heterogeneity (A) Unsupervised clustering analysis (hkmeans method; k = 3) obtaining three mutational scenarios from the scaled mutational signatures exposure and complex rearrangements. All mutational signatures from different variant types and complex rearrangements detected in our cohort are included in the analysis. Color scale indicates frequency. (B) Summary of the defining features for each of the three mutational scenarios/clusters. Color gradation corresponds to clustering distance. (C) Summary of the three mutational scenarios described in our neuroblastoma cohort, including different characteristic features such as mutational signatures, complex rearrangements, mutational processes, and risk classification correspondence associated with each of them. CRs, complex rearrangements. (D) Univariate Cox proportional hazards model. Forest plot shows the proportional risk of the three mutational scenarios and the three neuroblastoma risk groups. (E) Kaplan-Meier survival curves showing the clinical impact of the three mutational scenarios and the three neuroblastoma risk groups. (p = 0.00087 and p < 0.0001, respectively by log-rank test). Colors on Kaplan-Meier plot display each condition. All analyses were performed in the discovery cohort (n = 114).

We hypothesized that the co-occurrence of complex variant patterns and mutational signatures from independent variant types with similar molecular etiologies may be linked to the clinical heterogeneity observed in patients suffering from neuroblastoma. Unsupervised clustering based only on the frequency of the different types of mutational signatures (SNV-, indel-, CNA-, and SV-based) and complex rearrangement patterns grouped tumors into three different clusters, which we termed mutational scenarios (Figures 6A–6C and Table S7). In line with our hypothesis, mutational scenarios exhibited concordance of more than 80% with neuroblastoma risk group classification. Mutational scenario #1 was enriched in high-risk *MYCN*-amplified neuroblastomas and was defined by footprints of DNA damage by ROS (SBS18), replication slippage (ID1/ID2), replication stress (CX5/CX11), and SV-based signature SV3/R6b, association with clustered SVs, and focal oncogene amplification in the form of ecDNA. This nominates replication-associated mutagenesis as the underlying mechanism of variants occurring in this risk group, e.g., ecDNA (Figures 6A–6C). The second mutational scenario (scenario #2) was characterized by signs of impaired HRR (SBS3/ID6/CX3) and single base deletions (ID9). It was associated with a higher variety of linear chromosomal complex rearrangements such as CnCs, and TICs (SV5). This mutational scenario was enriched in high-risk non-*MYCN*-amplified neuroblastomas, suggesting that the high number of CNAs and SVs found in this risk group might be caused by the portrayed HRR deficiency, which is in line with recent reports in other cancer types.^50^ Supporting these findings, non-*MYCN*-amplified patients showed an enrichment in mutations affecting genes from the HRR pathway such as *BRIP1* and *RAD51C* (Figures 3D and 3E; p = 2.26 × 10^−4^). Interestingly, the presence of ecDNA was nearly mutually exclusive, with defective homologous recombination-associated signatures defining scenario #2 (Figures 3A and 3B). Consistently, ecDNA-harboring, *MYCN*-amplified neuroblastomas exhibited the lowest HRDetect probability scores, an alternative measure of HRR activity,^51^ across our cohort, with a median score of 1% and no patient with a score >70% (Figures 3A, 3C, and 3E; Table S8). Scenario #3 was linked to high prevalence of clock-like mutations (SBS5), topoisomerase-associated mutational activity (ID4), small simple deletions (SV1), and signs of chromosome mis-segregation (CX1/CX14). This mutational scenario was enriched in non-high-risk neuroblastomas. Concordance between mutational scenarios and clinical risk groups was also reflected by overall survival rates (e.g., non-high-risk and scenario #3 patients; Figures 6D and 6E; p = 8.7 × 10^−4^ and p < 1 × 10^−4^, respectively, by log-rank test). Thus, clinical neuroblastoma heterogeneity is significantly associated with risk-group-defining mutational footprints, which not only offers new insights into the etiology of disease group-defining genomic variants but also raises the possibility that differences in mutational processes during malignant transformation and tumor progression may contribute to inter-tumor phenotypic and clinical differences.

## Discussion

This study analyzed neuroblastoma genomes from clinically heterogeneous patients and classified the mutational processes and genomic rearrangement patterns based on all genomic variant classes. This led to the identification of three distinct mutational scenarios defined by the co-occurrence of mutational signatures and complex rearrangement patterns associated with similar mutational processes independent of variant classes. Scenario #1 was driven by DNA damage caused by ROS, replication slippage, and stress and was enriched in high-risk *MYCN*-amplified, ecDNA-harboring patients with low overall survival. Scenario #2 was driven by HRR-associated signatures and characterized by linear, chromosomal, complex rearrangements, frequently observed in high-risk non-*MYCN*-amplified patients with low survival. Scenario #3 was characterized by footprints of chromosome mis-segregation, topoisomerase activity, and high presence of clock-like signature SBS5, and was enriched in non-high-risk patients exhibiting good prognosis. Our findings demonstrate that clinical neuroblastoma heterogeneity is associated with differences in mutational footprints across genomic variant classes, offering a new perspective on the mutational processes contributing to neuroblastoma genesis and evolution.

Even though the identified mutational scenarios are linked to specific mutational processes,^36^^,^^37^^,^^52^^,^^53^^,^^54^^,^^55^^,^^56^ what causes these processes to be active in neuroblastoma currently remains unknown. Some molecular features in neuroblastomas can activate mutational processes. For example, MYCN can induce ROS, replication stress, and fork stalling.^57^^,^^58^ Thus, high *MYCN* expression in neuroblastomas harboring *MYCN* amplifications may explain increased footprints of ROS, replication slippage, and stress observed in mutational scenario #1, which was enriched in *MYCN*-amplified neuroblastomas. Recent reports suggest that the presence of ecDNA itself may cause high replication stress in cancer cells,^59^ indicating that some of the complex variants observed may themselves fuel mutational activity.

Deficiencies in HRR can cause the generation of simple and complex SVs,^60^ such as BFBs and chromothripsis.^61^ For example, mutations in *BRCA1/2* resulting in HRR deficiency are associated with high prevalence of certain SVs, termed *BRCA*ness.^62^ Although *BRCA1/2* mutations are not frequent in neuroblastoma,^63^ we identified mutational signatures related to HRR deficiency in high-risk non-*MYCN*-amplified patients. These tumors were also marked by higher homologous recombination deficiency (HRD) probability scores (HRDetect) and higher prevalence of mutated HRR genes, including the ones in the PROfound clinical trial.^64^ Thus, alterations other than *BRCA1/2* mutations may cause the apparent HRR deficiency in neuroblastomas. In line with our work, recent pediatric pan-cancer studies also observed signatures associated with HRR deficiency in neuroblastoma.^10^ The high prevalence of these footprints in high-risk compared to non-high-risk patients indicates that HRR deficiency may not only represent a mere feature of these tumors but could also contribute to their aggressive clinical behavior, similar to what was observed in HRR-deficient pancreatic cancers and gliomas.^65^ This may be especially relevant for future clinical trial designs, as HRR deficiency is associated with altered response to certain chemotherapeutic agents^66^ as well as increased sensitivity to pharmacological PARP (poly(ADP-ribose) polymerase) trapping.^67^ Thus, the identification of mutational patterns associated with HRR deficiency in high-risk neuroblastomas may have important implications for neuroblastoma risk and therapy stratification.

Changes in mutational process activities can inform the causes of tumor evolution.^68^^,^^69^ Our study revealed that in high-risk neuroblastomas ROS and HRR deficiency predominantly contribute to mutagenesis in early tumor development, while replication-associated mutations are accumulated throughout evolution. This indicates that the difference between high-risk and non-high-risk neuroblastomas may already be determined by the mutagenic processes involved in their early development and/or the initial transformation of their non-cancerous cell of origin.

We also provide a complete catalog of recently described complex variant patterns^17^ in neuroblastoma, including chromothripsis, BFBs, ecDNA, CnCs, chromoplexy, TICs, rigma, pyrgo, Dup-Trp-Dup, and Dup-invDup, and reveal their distinct co-occurrence patterns with mutational signatures, indicating differences in the mutagenic processes active during their generation. Moreover, we linked *de novo* signature SV5 with TICs, suggesting that recently topographically defined variant types can be reflected by novel SV signatures.

Extrachromosomal DNA is one of the most prevalent genomic alterations in cancer.^70^ We and others recently demonstrated that it not only serves as a vehicle for oncogene amplification^30^ but is also the most recurrent site of complex structural rearrangements.^16^^,^^30^^,^^31^ Indeed, footprints of BFBs, chromothripsis, and kataegis on ecDNA in other cancer entities nominated these processes as mechanisms of ecDNA generation.^32^^,^^33^ In contrast, we only observed signs of BFBs and chromothripsis on ecDNA in a subset of cases and did not observe footprints indicative of APOBEC3-driven kataegis to the same extent as observed elsewhere.^71^ Based on our most recent observations in single neuroblastoma cells^72^ and consistent with our mutational signature co-occurrence analyses, we propose that other processes such as replication slippage may create structurally simpler ecDNA in neuroblastomas in which BFBs and chromothripsis were not detected. Such simple ecDNAs may then evolve and gain in complexity through further mutagenesis. For example, recently described ecDNA reintegration and circular recombination may contribute to ecDNA evolution and result in similarly complex structures as those created through BFBs or chromothripsis.^30^^,^^33^ The relative contributions of these different mechanisms in the structural shaping of ecDNA are still largely unresolved. Combined detection of the mutational scenarios defined in this study with longitudinal single-cell sequencing may facilitate the investigation of these open questions.

In summary, our study provides a comprehensive classification of active mutational processes in neuroblastoma, offering new insights into the origin of genomic alterations involved in neuroblastoma genesis and progression. The three mutational scenarios presented here not only refine our understanding of neuroblastoma’s clinical heterogeneity but may also improve our understanding of how mutational processes contribute to the generation of different variant classes in cancer in general.

### Limitations of the study

While the unsupervised clustering of neuroblastoma patients in the three mutational scenarios was in high concordance (>80%) with the clinical risk group classification, we encountered some patients who were considered as outliers. These patients, although clinically classified in a specific risk group, showed genomic features associated with other risk groups. Owing to the limitation in sample size, we were not able to draw significant conclusions that could explain these discrepancies between the genomic and clinical features. We also did not include transcriptomic data in this study; such data could be helpful in future projects for the understanding of the mentioned outlier divergence and the contribution of specific gene expression to the mutational scenarios that we describe.

## STAR★Methods

### Key resources table

**Table:** 

REAGENT or RESOURCE	SOURCE	IDENTIFIER
**Deposited data**		

Neuroblastoma whole-genome sequencing data	European Genome-Phenome Archive https://ega-archive.org/	EGA: EGAS00001001308,^34^ EGA: EGAS00001004022,^30^ EGA: EGAS00001006983, EGA: EGAS00001007016, and EGA: EGAS00001007019
Original code for all the analyses	Henssen lab	Github: https://github.com/henssen-lab/mutsignsNBLpaper
Raw data results	This paper	Zenodo: https://doi.org/10.5281/zenodo.8032024

**Software and algorithms**		

FastQC v.0.11.8	Babraham bioinformatics^73^	https://www.bioinformatics.babraham.ac.uk/projects/fastqc/
BWA-MEM v.0.7.15	Li et al.^74^	https://github.com/lh3/bwa
SAMtools v.1.10	Danecek et al.^75^	https://github.com/samtools/samtools
Biobambam v.2.0.87	Tischler et al.^76^	https://gitlab.com/german.tischler/biobambam2
Mutect2 v.4.1.8.1	McKenna et al.^77^	https://github.com/broadinstitute/gatk/releases
GATK v.4.1.9.0	McKenna et al.^77^	https://github.com/broadinstitute/gatk/releases
bedtools v.2.29.2	Quinlan et al.^85^	https://bedtools.readthedocs.io/en/latest/index.html
ASCAT v.2.6	Van Loo et al.^81^	https://github.com/VanLoo-lab/ascat
Novobreak v.1.1.3	Chong et al.^80^	https://github.com/czc/nb_distribution
SvABA v.1.1.0	Wala et al.^79^	https://github.com/walaj/svaba
Delly2 v.0.7.7 and v.0.8.1	Rausch et al.^78^	https://github.com/dellytools/delly
Ensembl VEP v.102.0	McLaren et al.^86^	https://www.ensembl.org/info/docs/tools/vep/index.html
mutSignatures v.2.1.1	Fantini et al.^87^	https://github.com/dami82/mutSignatures
YAPSA v.1.16.0	Daniel Huebschmann et al.^88^	https://github.com/HiDiHlabs/YAPSA
Palimpsest v.2.0.0	Letouzé et al.^89^	https://github.com/FunGeST/Palimpsest
CNA signatures (no name)	Drews et al.^8^	https://github.com/markowetzlab/Drews2022_CIN_Compendium
JaBba v.1.0	Hadi et al.^17^	https://github.com/mskilab/JaBbA
Amplicon Architect v.1.2	Deshpande et al.^91^	https://github.com/virajbdeshpande/AmpliconArchitect
Shatterseek v.0.5	Cortés-Ciriano et al.^92^	https://github.com/parklab/ShatterSeek
gGnome v.0.1	Imieliński, M.	https://github.com/mskilab/gGnome
AmpliconClassifier v.0.4.6	Deshpande et al.^16^	https://github.com/jluebeck/AmpliconClassifier
MuSE2.0 v.1.0rc	Fan et al.^81^	https://github.com/danielfan/MuSE
Pindel v.0.2.5b9	Ye et al.^82^	https://github.com/genome/pindel
Battenberg v.2.2.9	Nik-Zainal et al.^84^	https://github.com/Wedge-lab/battenberg
TrackSigFreq	PCAWG Evolution and Heterogeneity Working Group et al.^41^	https://github.com/morrislab/TrackSigFreq
R v.4.0.3 with packages:		N/A
ComplexUpset v.1.2.1	Lex et al.^93^	https://krassowski.github.io/complex-upset/index.html
corrplot v.0.90	Taiyun et al.^96^	https://github.com/taiyun/corrplot
dplyr v.1.0.3	Wickham et al.^99^	https://dplyr.tidyverse.org/
GenomicRanges v.1.42.0	Lawrence et al.^97^	https://bioconductor.org/packages/release/bioc/html/GenomicRanges.html
ggplot2 v.3.3.5	Wickham et al.^98^	https://ggplot2.tidyverse.org/
gUtils v.0.2.0	http://mskilab.com/gGnome/tutorial.html	https://github.com/mskilab/gUtils
IRanges v.2.24.0	Lawrence et al.^97^	https://bioconductor.org/packages/release/bioc/html/IRanges.html
ComplexHeatmap v.2.4.2	Gu et al.^101^	https://bioconductor.org/packages/release/bioc/vignettes/ComplexHeatmap/inst/doc/complex_heatmap.html
reshape v.0.8.8,	Wickham et al.^102^	https://cran.r-project.org/web/packages/reshape/index.html
pairwiseComparisons v.3.1.2	Patil et al.^103^	https://cran.r-project.org/src/contrib/Archive/pairwiseComparisons/
ggstatsplot v.0.6.8	Patil et al.^104^	https://indrajeetpatil.github.io/ggstatsplot/
Hmisc v.4.4.2,	Harrell et al.^105^	https://cran.r-project.org/web/packages/Hmisc/index.html
BSgenome.Hsapiens. UCSC.hg19	Team TBD	https://bioconductor.org/packages/release/data/annotation/html/BSgenome.Hsapiens.UCSC.hg19.html
survival v.3.2–11	Therneau et al.^95^	https://cran.r-project.org/web/packages/survival/index.html
survminer v.0.4.9	Kassambara et al.^100^	https://cran.r-project.org/web/packages/survminer/index.html
signature.tools.lib v2.1.2	Degasperi et al.^9^	https://github.com/Nik-Zainal-Group/signature.tools.lib
factoextra v1.0.7	Kassambara et al.^94^	https://cran.r-project.org/web/packages/factoextra/index.html

### Resource availability

#### Lead contact

Further information and requests for resources should be directed to and will be fulfilled by the lead contact, Anton G. Henssen (henssenlab@gmail.com).

#### Materials availability

This study did not generate new unique reagents.

### Experimental model and study participant details

This study comprised the analyses of tumor and blood samples of 150 human patients diagnosed with neuroblastoma between 1991 and 2016. All stages, sex and age were included in this study (Figure 1). All samples were collected at diagnosis from untreated patients. Patients were registered and treated according to the trial protocols of the German Society of Pediatric Oncology and Hematology (GPOH). This study was conducted in accordance with the World Medical Association Declaration of Helsinki (2013) and good clinical practice; informed consent was obtained from all patients or their guardians. The collection and use of patient specimens was approved by the institutional review boards of Charité-Universitätsmedizin Berlin and the Medical Faculty, University of Cologne. Specimens and clinical data were archived and made available by Charité-Universitätsmedizin Berlin or the National Neuroblastoma Biobank and Neuroblastoma Trial Registry (University Children’s Hospital Cologne) of the GPOH.

### Method details

#### Sequencing data

This study is based on the analysis of two cohorts: (1) Discovery cohort, and (2) Validation cohort.

##### Discovery cohort

120 neuroblastoma primary tumor and matching control samples. Those samples are divided into two datasets. The first one, sequenced in Cologne, was completed in 2015,^34^ and it is composed of WGS from 56 primary tumor and matching blood control samples (Illumina HiSeq). It was downloaded (https://www.ebi.ac.uk/ega/) under accession number EGA: EGAS00001001308.^34^ The second one, sequenced at the German Cancer Research Center, was completed in 2017–2019, and it includes WGS of 64 primary tumor and matching control samples (Illumina HiSeq X Ten). The data of 53 out of the total 64 patients was publicly available and downloaded (https://www.ebi.ac.uk/ega/) under accession number EGA: EGAS00001004022.^30^ The data of the 11 remaining patients is now available (https://www.ebi.ac.uk/ega/) under accession number EGA: EGAS00001006983. The quality of the raw data was assured using FastQC.^73^ Reads were 3′ trimmed for both quality and adapter sequences, with reads removed if the length was shorter than 20 nucleotides. Burrows–Wheeler Aligner MEM^74^ v.0.7.15 with default parameters was used to align^75^ the reads to human reference assembly hg19. PCR and optical duplicates were marked with bammarkduplicates2 from BIOBAMBAM2^76^ v2.0.87 (https://github.com/gt1/biobambam2). Six patients have been excluded from the analyses due to an abnormally high number of SVs detected (CB2044, NBL47, NBL49, NBL50, NBL53, NBL54). They present in average more than 5-fold SVs than the rest of the cohort (median of SVs of the cohort: 14 SVs per patient; median of SVs of the excluded patients: 70.5 SVs per patient).

##### Validation cohort

This dataset was completed in January 2022, and it is composed of ultra-deep WGS (x200) from 39 primary tumor and matching control samples (adrenal gland, blood, fat tissue, lymph node, muscle, skin) (Illumina NovaSeq S4). The data is available (https://www.ebi.ac.uk/ega/) under accession numbers EGA: EGAS00001007016 and EGA: EGAS00001007019. The quality of the raw data was assured using FastQC.^73^ Reads were 3′ trimmed for both quality and adapter sequences, with reads removed if the length was shorter than 20 nucleotides. Burrows–Wheeler Aligner MEM^74^ v.0.7.15 with default parameters was used to align^75^ the reads to human reference assembly hg19. PCR and optical duplicates were marked with bammarkduplicates2 from BIOBAMBAM2^76^ v2.0.87 (https://github.com/gt1/biobambam2). Three patients have been excluded from the analyses due to a contamination on normal sample (A06R-NFBQNJ, A06R-NFVDJM, A06R-GNBIPE).

#### Variant calling

##### SNVs and indels

Somatic single-nucleotide variants and small insertions and deletions were detected using Mutect2 v.4.1.8.1 from the GATK^77^ software package, with standard parameters according to GATK best practices recommendations. Variants were filtered using recommended filtering parameters, removing all germline mutations using a panel of normals.

##### SVs

In matched normal and tumor samples, somatic structural variants were called using three different variant callers with their corresponding complementary methodologies: Delly2^78^ v0.7.7, SvABA^79^ v.1.1.0, and Novobreak^80^ v.1.1.3 with default parameters. To increase specificity, we followed the standard practices for SV consensus calling and intersected all the variants from the three callers keeping the ones detected at least in two of them. An exception has been made for insertions since we only identified them using SvABA. Variants with both breakpoints within a window of 500 bp were collapsed. To increase sensitivity in our consensus approach, each variant included in the final set passed all the standard filters in at least one of the required two callers to be considered in our analysis, not requiring the passing of all the standard filters in both callers.

##### CNAs

Allele-specific copy-number (CN) profiles were generated from tumor and normal B allele frequencies (BAFs) and LogR values using ASCAT^81^ v2.6 with parameters adjusted for sequencing data input (segmentation penalty = 200 and aspcf function gamma = 1). The segmentation procedure from ASCAT was replaced with a custom implementation that only considers BAFs to determine start and endpoints of segments but still estimates the segment’s coverage using the log coverage ratios. This approach avoids potential oversegmentation due to noisy LogR values. All CN profiles were inspected manually for quality control. For samples with an estimated tumor purity, below 60% CN calling was rerun with adjusted purity and ploidy values that were manually selected after inspection of the sunrise plots and in agreement with pathology estimates of tumor purity. We assigned different copy number states to all profiles considering the local copy number and overall ploidy estimates. CN gain was defined as log2((Cnmajor + Cnminor)/ploidy) > 0.3; CN loss as log2((Cnmajor + Cnminor)/ploidy) < −0.3. CN amplifications and homozygous losses have been determined following COSMIC CNA parameters (https://cancer.sanger.ac.uk/cosmic/help/cnv/overview). In the case of amplifications, we added an additional filter of log2((Cnmajor + Cnminor)/ploidy) > 1.25 to increase specificity. For three patients no CNA results could be obtained (NBL31, NBL36, and NBL61).

##### Variant calling in the validation cohort

Somatic SNVs were detected using MuSE2.0^82^ v1.0rc (https://github.com/wwylab/MuSE) in two-steps: Muse Sump and Muse call, using recommended parameters. SNVs were filtered using tier-based cutoffs from a sample-specific model computed by the method, removing Tier5 class. Indels were called using Pindel^83^ v 0.2.5b9. with standard parameters and filtered following the next parameters; *VAF=0.2, cov = 20, hom = 6*. Somatic SVs were detected using Delly2^78^ v0.8.1 with default parameters. SVs were processed and filtered using recommended filtering parameters, removing contamination and variants <50bp in length. Allele-specific copy-number (CN) profiles were generated using Battenberg^84^ v2.2.9 with default parameters. All the reference files used on general static were downloaded from (https://ora.ox.ac.uk/objects/uuid:2c1fec09-a504-49ab-9ce9-3f17bac531bc) following the recommendations by the developers of the method.

#### Identification of variants affecting genes and chromosome arms

##### Chromosomal arms

Gains and losses of chromosomal arms have been determined intersecting the coordinates of the chromosomal regions with the CNA gains and losses, using bedtools^85^ v2.29.2 with parameters *-wo -f 1 -F 0.60* (https://bedtools.readthedocs.io/). We considered an overlap of the whole CNA with at least 60% of the chromosomal arm.

##### Genes

We predicted the variant effect for SNV and indel calls using Ensembl VEP^86^ (Variant Effect Predictor) v.102.0. Only SNVs and indels classified as *missense* and *stop gained* overlapping genes, were considered. For SV calls, we considered all the genes affected by SV breakpoints. We classified them in two types: (1) SV close to gene, defined as genes with at least one SV breakpoint at a distance of +/− 20 kb; (2) SV within gene, defined as genes overlapping at least one SV breakpoint. For CNA calls, we considered the genes overlapping amplifications or homozygous deletions.

#### Mutational signatures analyses

The mutational signatures analyses have been performed using the same methodology for both the discovery and validation cohorts.

##### SNV signatures (SBS)

To retrieve mutational signatures related to somatic single-nucleotide variants operating in neuroblastoma, we used the R package mutSignatures^87^ v.2.1.1. We started pre-processing all the SNVs in coding and non-coding regions detected in our patients. We applied a filtering step for non-SNV variants, followed by the tri-nucleotide context extraction for each of the variants which are classified in 96 different mutation types, or features, and counted across samples. Next, we ran the *de-novo* signature extraction step where non-negative matrix factorization (NMF) is performed. Complying with the pipeline recommendations, we computed 500 iterations, first, evaluating different choice of ranks/number of signatures: *k=4*, *k=5*, and *k=6*. We chose *k=4*, resulting in four *de-novo* signatures, based on the presence of clusters with consistent non-negative high silhouette scores. Then, we determined which known COSMIC v.3.2 signatures (94 SBS reference signatures; https://cancer.sanger.ac.uk/signatures/sbs/) matched our *de-novo* signatures, using a cosine similarity threshold of ≥0.85. In the deconvolution step, we estimated the absolute and relative exposure of the COSMIC mutational signatures in each of the patients. Next, to increase specificity and reduce the false positive assignment we filtered all signatures showing an absolute exposure lower than 5% of the total number of mutations in our cohort. Finally, we obtained four COSMIC signatures: SBS3, SBS5, SBS18, and SBS40. The same signatures were extracted in the validation cohort.

##### Indel signatures (ID)

Mutational signatures associated with small somatic insertions and deletions (<50 bp) have been extracted using the R package YAPSA^88^ v.1.16.0, which includes PCAWG/COSMIC indel signatures (18 ID reference signatures; https://cancer.sanger.ac.uk/signatures/id/), following the standard pipeline. This pipeline starts with a pre-processing step of all the indels detected in our patients, in which we annotate the sequence context 10bp downstream and 60bp upstream of each variant, the variant type, and the length. In line with the SNV signature analysis, indels are classified in 83 different classes of features, and counted across samples. Next, we computed the supervised mutational signature analysis step, based on the linear combination decomposition function (LCD), obtaining the exposure for each known mutational signature in each patient. Signature exposure per neuroblastoma risk group was estimated from the exposure per patient. In order to reduce false positive calls, recommended signature-specific cutoffs for the indel-based PCAWG signatures have been applied, using the determined value of *optimal cost factor = 3*. As an additional step to increase specificity, we filtered all signatures showing an absolute exposure lower than 5% of the total number of indels in our cohort. From this analysis, we have obtained six PCAWG/COSMIC indel signatures: ID1, ID2, ID4, ID6, ID8, and ID9. Applying the same methodology to the validation cohort we obtained: ID2, ID4, ID5, ID6, and ID9 indel signatures.

##### CNA signatures (CX)

To quantify the activity of copy number signatures in our samples, we used the 17 signatures that have been derived from over 6,000 tumors of the cancer genome atlas,^8^ following the methodology used by Drews et al. (17 CX reference signatures; https://github.com/markowetzlab/Drews2022_CIN_Compendium). Using the raw absolute copy number as obtained from ASCAT (for the discovery cohort) or Battenberg (for the validation cohort) as input, very copy number quiet samples are removed and first 43 features describing the fundamental features of the copy number profiles are extracted from the remaining profiles. Afterward the exposure for each previously described signature in each of our samples is calculated based on the posterior distribution of the extracted features. As an additional step to increase specificity, we filtered all signatures showing an exposure lower than 5% of the total exposure in our cohort. From this analysis we obtained eight CNA-based signatures: CX1, CX2, CX3, CX5, CX7, CX11, CX14, and CX15. Applying the same methodology to the validation cohort we obtained: CX1, CX2, CX3, CX4, CX6, CX14, and CX15 CNA signatures.

##### SV signatures (SV)

Mutational signatures associated with somatic structural variants have been extracted using the R package Palimpsest^89^ v.2.0.0 following the standard pipeline for this variant type. This pipeline starts with a pre-processing step in which we annotate the type (deletions, inversions, tandem-duplications, and translocations), size (<1 kb, 1–10 kb, 10–100 kb, 100 kb–1 Mb, 1–10 Mb, and >10 Mb), and clustered nature of rearrangements (distinguish between clustered – ≥ 10 breakpoints within a 1 Mb window – and non-clustered events). SVs are classified into 38 different classes of features and counted across samples. Next, we computed the *de-novo* signature extraction step, based on NMF. Complying with the pipeline recommendations, we computed 500 iterations with *num_of_sigs = auto* (the appropriate rank/number of signatures was estimated from NMF metrics such as the cophenetic distance, obtaining *k=5*). In the final step of the pipeline, we estimated the absolute and relative exposure of each signature in each sample. Following what we did for the other variant types to increase specificity we filtered all signatures showing an absolute exposure lower than 5% of the total number of mutations in our cohort. As a result, we obtained five *de novo* signatures. In order to evaluate if the *de novo* SV-based signatures found in neuroblastoma matched any of the 21 reference SV signatures from previous pan-cancer studies^9^ we used the *deconvolution_compare* function of Palimpsest obtaining cosine similarities between signatures. Using the same cosine similarity threshold from the SNV analysis (≥0.85) we identified two matches. *De novo* signatures SV2, and SV3 corresponded to reference signatures R6a (*MDM2*, *CDK4*, and 17q mutations), R6b (*MDM2*, and *CDK4* mutations).^9^ Although SV1 and R2 (*TP53* mutations) also showed a cosine similarity ≥0.85 we could not confirm the match due to mutational profile distances (high presence of small deletions < 1kb in SV1). In addition, we described three *de-novo* signatures that did not match any reference signature. We named them based on the prevalence of SVs in the different features classes: (1) SV1, small deletions; (2) SV4, large simple intra and interchromosomal SVs + clustered translocations; (3) SV5, medium size simple intra and interchromosomal SVs. Applying the same methodology to the validation cohort we obtained 5 *de novo* signatures, 3 of which present a cosine similarity >0.75 with the SV-based signatures from the discovery cohort.

From the patient’s exposure, we assessed the contribution of each of the different mutational signatures from the different variant types within neuroblastoma risk groups.

#### Subclonal signatures analysis

The analysis of subclonal signature trajectories has been performed with the method TrackSigFreq^41^ based on the observed density of mutation frequencies and changes in mutational signature activities. This method has been used instead of its predecessor, TrackSig,^90^ because it adds the advantage to identify distinct populations of mutations that share similar signature activities. TrackSigFreq has been executed using the SNV VCF file generated by MuSE2.0^82^ as described in the *variant calling* section, and copy number and purity information from Battenberg.^84^ We selected the mutational signatures detected by mutSignatures, as explained in the *mutational signatures analysis* section, to evaluate the changes in the trajectory of the mutational signature activity through the different cancer cell fractions. To avoid losing mutational signatures with low activity, we set the exposure filter to 0 to visualize the performance of all mutational signatures without restrictions. To compare the activity of the different signatures across the cancer cell fraction (CCF), we selected for each signature the exposures for the highest and lowest CCF, per patient. We used the same *statistical analysis* explained below, comparing the frequencies of signature activity per risk group, and per CCF.

#### Homologous recombination deficiency analysis

The analysis of homologous recombination deficiency has been performed using two complementary approaches: 1) presence of somatic mutations in HRR genes, and 2) HRDetect algorithm.

##### HRR genes

The presence of mutations in HRR genes has been evaluated using the set of somatic variants described in the *Variant calling* section above, including SNVs, indels, SVs, and CNAs. We predicted the variant effect for SNV and indel calls using Ensembl VEP^86^ (Variant Effect Predictor) v.102.0. Only SNVs and indels classified as *missense* and *stop gained* overlapping genes, were considered. For SV calls, we considered all the genes affected by: (1) SV close to gene; (2) SV within gene, defined in the *Identification of variants affecting genes and chromosome arms* section above. For CNA calls, we considered the genes overlapping amplifications or homozygous deletions. The set of HRR genes considered in this analysis correspond to 94 genes associated to the homologous recombination repair (HRR) pathway, including the 15 HRR genes from the PROfound clinical trial^64^ (*BRCA1*, *BRCA2*, *ATM*, *BRIP1*, *BARD1*, *CDK12*, *CHEK1*, *CHEK2*, *FANCL*, *PALB2*, *PPP2R2A*, *RAD51B*, *RAD51C*, *RAD51D*, and *RAD54L*). We used the mutational status of these genes to compute the correlation with the activity of mutational signatures and complex rearrangements in our data.

##### HRDetect analysis

For this analysis we used the *signature.tools.lib* R package^9^ (https://github.com/Nik-Zainal-Group/signature.tools.lib) for mutational signatures analysis, which includes the HRDetect pipeline that computes the HRDetect BRCAness probability score.^51^ Following the best practices specified by the authors, we used the tri-nucleotide context from our previous mutational signature analysis along with the COSMIC v.3.2 reference signature catalog. We also supplied the indel, SV, and CNA calling from the *Variant calling* step described above, to compute indels classification, rearrangements, and the copy number-based score HRD-LOH. Then the method extracts six features: (1) proportion of deletions at microhomology; (2) SBS3 exposure; (3) R3 exposure; (4) SBS5 exposure; (5) HRD LOH index; (6) SBS8 exposure. Finally, the function returns the HRDetect BRCAness probability score for all the samples. We used this probability score to compute the correlation with the activity of mutational signatures and complex rearrangements in our data, along with the evaluation of differences of BRCAness probability between risk groups.

#### Complex rearrangements calling and classification

To detect and reconstruct all linear and circular complex rearrangements types in neuroblastoma, we used three established complementary algorithms: JaBba^17^ v1.0, Amplicon Architect^91^ v.1.2, and the R package Shatterseek^92^ v.0.5.

##### JaBba

JaBba^17^ has been run using standard parameters and following the best practices pipeline, including the pre-processing steps using fragCounter and dryclean to correct for GC content and mappability and denoise the coverage data using a panel of normals. From these steps, we obtained the coverage input that we use along our SV calls to run the pipeline. Then, following what has been done in previous studies,^17^ the output from JaBba was run into gGnome v.0.1 to classify the different complex SVs into rigma, pyrgo, TIC, chromoplexy, chromothripsis, BFB, double minute (DM), and tyfonas.

##### AmpliconArchitect

We used the copy number profiles obtained from ASCAT and the bam files of all tumor samples as input to PrepareAA^91^ (https://github.com/jluebeck/PrepareAA), a wrapper function that handles the preprocessing and execution of AmpliconArchitect v.1.2.

We predicted the types of amplifications present in Amplicon Architect’s^91^ output using AmpliconClassifier^16^ v.0.4.6 with standard parameters except for –force = TRUE. With this method we classified circular amplicons in ecDNA and BFB, and linear amplicons in complex non-cyclic amplicons (CnC).

##### ShatterSeek

ShatterSeek^92^ has been run from CNA and SV calls using standard parameters to detect chromothripsis. The filtering steps presented in the tutorial to obtain high confidence calls have been followed: at least 6 interleaved intrachromosomal SVs, 7 contiguous segments oscillating between 2 CN states, significant fragment join tests, and either significant chromosomal enrichment or exponential distribution of breakpoints test. In order to include chromothriptic candidates in our results that might affect highly amplified regions, we also called low-confidence chromothripsis having at least 6 interleaved intrachromosomal SVs and 7 contiguous segments. In these cases, a final step of visual inspection was performed.

The three complex rearrangement calling pipelines have been run using the hg19 reference genome. The consensus variant calling has been used for this analysis. Complex rearrangements detected by at least one method were considered. We evaluated each of the overlapping complex rearrangements per patient. All overlapping complex rearrangements detected in the same patient showing similar overlapping segments and junctions have been collapsed, keeping the one with most informative classification.

##### *Clustered rearrangements* identification

With the aim of classifying all those clustered SVs not falling into the known complex rearrangement categories we developed a method following the clustering definition used in SV-based mutational signature analysis. Considering the consensus SV calling, we classified as clustered rearrangements the events exhibiting ≥10 SV breakpoints within a 10 Mb window of the genome. All clustered rearrangements overlapping known complex rearrangements detected in our cohort were filtered out.

#### Functional analysis of complex rearrangements

Co-occurrence of the different complex rearrangement types was computed considering the presence of the different rearrangement classes per patient. The figure has been created using the R package ComplexUpset^93^ v.1.2.1 (https://github.com/krassowski/complex-upset).

The ratio of SVs involved in complex rearrangements has been extracted establishing the ratio of the number of SVs overlapping segments or junctions of those rearrangements per patient.

For all detected complex rearrangement class in our cohort, we obtained the coordinates of each complex SV. Then, using 1 Mb windows of the genome, we plotted the density of regions affected by complex rearrangements in each chromosome across the whole human genome.

Using the coordinates information of complex rearrangements, we evaluated the overlap of the segments and junctions of these clustered events with cancer-related genes and DNA repair genes using *intersectBed* function from bedtools^85^ v2.29.2 with parameters *-wo* (https://bedtools.readthedocs.io/). We performed this analysis for each complex rearrangement class separately to obtain which genes were affected by each class differentially.

#### Kataegis analysis

We considered a cluster of kataegis when we called 6 or most consecutive SNVs with an intermutation distance of < 1kb. The same method has been used in previous studies.^32^

#### Mutational scenarios analysis

The three mutational scenarios are generated using a hybrid between hierarchical and k-means unsupervised clustering methodology (*hkmeans* from the *factoextra* R package^94^). We set the rank/number of clusters at *k=3*, after evaluation using *fviz_nbclust*, from the same package, which determines and visualize the optimal number of clusters using different methods such as gap statistics. The information fed to the clustering method was the scaled frequency of the different types of mutational signatures (SNVs, indels, CNAs, and SVs) and complex rearrangements for each single patient. No signatures were excluded. No risk group classification, mutational status of known driver genes (i.e., *ALK*, *MYCN*, *ATR*, etc.) or presence of neuroblastoma-associated chromosomal arms rearrangements (i.e., 17q, 11p, 1p, etc.) was used to compute the clustering. From the mutational signatures and complex rearrangements data, we obtained 3 different clusters which we named mutational scenarios, which when compared to the clinical risk group classification, showed a concordance of more than 80% of the samples. Scenario #1 was enriched in high-risk *MYCN*-amplified patients (20 HR MNA, 3 HR non-MNA, 1 non-HR), scenario #2 in high-risk non-*MYCN*-amplified patients (1 HR MNA, 33 HR non-MNA, 7 non-HR), and scenario #3 in non-high-risk patients (3 HR MNA, 5 HR non-MNA, 41 non-HR). From the results of the hybrid clustering analysis, we were able to extract the mean clustering distances for each feature (mutational signatures and complex rearrangements) per scenario. Using this information, and considering a distance threshold of 0.3 to contemplate that the feature of interest has a moderate/strong association, we identified the defining features for each scenario.

### Quantification and statistical analysis

All comparisons between distributions in the different neuroblastoma risk groups were made using the non-parametric Wilcoxon rank-sum test. To assess if there are differences between risk groups, we used the non-parametric Kruskal-Wallis test. The relationship between different variables such as signatures, complex rearrangements, and mutated genes has been calculated using Spearman’s correlation coefficient. All statistical analyses have been corrected by multiple testing when applicable using the false discovery rate (FDR) correction. All p values in the main text have been obtained using the Wilcoxon rank-sum test unless stated otherwise. The significance level has been established at p < 0.05.

Log rank tests were used for survival analysis across subgroups. To assess the clinical impact of the three defined mutational scenarios, we stratified our patients by scenario and clinical risk group classification. With this methodology we were able to evaluate the differences in overall survival between scenarios and risk groups. The subgroups for the analysis were: mutational scenario #1 (n = 24; 20 HR MNA, 3 HR non-MNA, 1 non-HR), mutational scenario #2 (n = 41; 1 HR MNA, 33 HR non-MNA, 7 non-HR), mutational scenario #3 (n = 49; 3 HR MNA, 5 HR non-MNA, 41 non-HR), high-risk MYCN-amplified patients (n = 24), high-risk non-MYCN-amplified patients (n = 41), and non-high-risk patients (n = 49). To assess the clinical impact of complex rearrangements in this tumor, we stratified our patients by the presence/absence of linear and circular complex SVs. The subgroups for the analysis were: Linear comp. rearrang.^-^/ecDNA^−^ (n = 63), Linear comp. rearrang.^+^/ecDNA^−^ (n = 26), Linear comp. rearrang.^-^/ecDNA^+^ (n = 15), and Linear comp. rearrang.^+^/ecDNA^+^ (n = 10).

To assess the hazard risk associated to the mutational scenarios compared to neuroblastoma risk group classification, we computed two univariates Cox proportional hazards regression analysis using the *coxph* function from the *survival* R package.^95^ Same subgroups used for the survival analysis, defined above, were used here. Hazard ratios, give the proportional clinical risk of belonging to each of the subgroups, including upper and lower 95% confidence intervals. Scenario #3 and non-high-risk patients have been used as reference in this analysis. Statistical significance was assessed through the Wald statistic value for each variable and through the Log rank test for the global statistical significance of the model.

Different additional R packages^96^^,^^97^^,^^98^^,^^99^^,^^100^^,^^101^^,^^102^^,^^103^^,^^104^^,^^105^ (see KRT) have been used to compute and plot the results from these analyses.

## Data Availability

• The WGS data that support the findings of this study have been deposited with the European Genome-phenome Archive (https://www.ebi.ac.uk/ega/) under accession nos. EGA: EGAS00001001308,^34^ EGA: EGAS00001004022,^30^ EGA: EGAS00001006983, EGA: EGAS00001007016, and EGA: EGAS00001007019 and are publicly available as of the date of publication. • All original code used to analyze the sequencing data, perform the statistical analysis, and generate the plots have been deposited and is publicly accessible in GitHub Github: https://github.com/henssen-lab/mutsignsNBLpaper as of the date of publication. • All the raw data required to support the conclusions reported in this manuscript, including the results from variant calling and mutational signature analysis, is available in Zenodo under DOI: Zenodo: https://doi.org/10.5281/zenodo.8032024. • Any additional information required to reanalyze the data reported in this paper is available from the lead contact upon request.

## Supplemental information

Document S1. Figures S1–S5

Table S1. Line-level data describing all the patients in this study, from both the discovery (n = 114) and validation (n = 36) cohorts, related to Figure 1 Table with line-level data including patient ID, age, cohort, risk group, sex, stage of the disease, treatment, and time of sample collection for each of the 150 neuroblastoma patients analyzed in this study.

Table S2. Reference etiologies of all the mutational signatures (SNV-, indel-, SV-, and CNA-based) identified in both the discovery (n = 114) and validation (n = 36) cohorts, related to Figures 2, 3, and 6 The etiologies of each mutational signature have been obtained from the databases COSMIC (SNV- and indel-based signatures; https://cancer.sanger.ac.uk/signatures/) and SIGNAL (SV-based signatures; https://signal.mutationalsignatures.com/) and from Drews et al.^8^ (CNA-based signatures). The main etiologies are underlined for each signature. The asterisks (∗) show which signatures have been extracted only in the evaluation cohort.

Table S3. Cosine similarity scores for SNV- and SV-based *de novo* extracted signatures in both the discovery (n = 114) and validation (n = 36) cohorts, related to Figure 2 For the signatures extracted *de novo* (SNV- and SV-based), we compared their pattern with reference signatures from COSMIC and SIGNAL databases obtaining the cosine similarity scores included in this table. The last three columns correspond to the comparison between SV-based signatures from both cohorts (see also Figures S3E and S3F).

Table S4. Evaluation of the differences in mutational activity between risk groups for all independent variant types in both discovery (n = 114) and evaluation (n = 36) cohorts, related to Figures 2A–2D The exposure frequencies depicted in Figures 2A–2D are specified. Neuroblastoma risk groups correspond to HR MNA (high-risk *MYCN*-amplified), HR non-MNA (high-risk non-*MYCN*-amplified), and non-HR (non-high-risk). Significance is established at p < 0.05.

Table S5. Evaluation of the differences in SNV-based mutational signature activity between risk groups in high/low cancer cell fraction (CCF), related to Figure 2F The mutational signature activity has been obtained using TrackSigFreq in the validation cohort (n = 36). CCF, cancer cell fraction. Neuroblastoma risk groups correspond to HR MNA (high-risk *MYCN*-amplified), HR non-MNA (high-risk non-*MYCN*-amplified), and non-HR (non-high-risk). Significance is established at p < 0.05.

Table S6. MYCN-amplification validation through FISH, compared with MYCN-including ecDNA inferred with computational methods in the discovery cohort (n = 114), related to Figures 3A, 3B, 4, and 5 ecDNA, circular extrachromosomal DNA; HSR, homogeneously staining region.

Table S7. Evaluation of the differences in complex rearrangements between risk groups in the discovery cohort (n = 114), related to Figure 4D The frequencies depicted in Figure 4D are specified along with the counts illustrated by Figure S5A. Neuroblastoma risk groups correspond to HR MNA (high-risk *MYCN*-amplified), HR non-MNA (high-risk non-*MYCN*-amplified), and non-HR (non-high-risk). Significance is established at p < 0.05. CR, complex rearrangement.

Table S8. Evaluation of the clustering of patients in the different mutational scenarios, related to Figure 6A The concordance coefficient (in percentage) established the proportion of patients that have been correctly classified according to the mutational scenario-risk group association: high-risk *MYCN*-amplified patients in scenario #1, high-risk non-*MYCN*-amplified patients in scenario #2, and non-high-risk patients in scenario #3.

Table S9. Homologous recombination deficiency analysis in the discovery cohort (n = 114), related to Figures 3A and 3C–3E Spearman correlation between mutational signatures activity, complex rearrangements frequency, and mutations in homologous recombination repair genes and HRDetect probability score.

Document S2. Article plus supplemental information
