# Supplementary material for: Mutational topography reflects clinical neuroblastoma heterogeneity
Source: Cell Genom. 2023 Sep 7;3(10):100402. doi: 10.1016/j.xgen.2023.100402 (PMC10589636; doi:10.1016/j.xgen.2023.100402)
Supplement: Document S1. Figures S1–S5 [file mmc1.pdf]

Cell Genomics, Volume *3*

Supplemental information

Mutational topography reflects clinical neuroblastoma heterogeneity

Elias Rodriguez-Fos, Mercè Planas-Fèlix, Martin Burkert, Montserrat Puiggròs, Joern Toedling, Nina Thiessen, Eric Blanc, Annabell Szymansky, Falk Hertwig, Naveed Ishaque, Dieter Beule, David Torrents, Angelika Eggert, Richard P. Koche, Roland F. Schwarz, Kerstin Haase, Johannes H. Schulte, and Anton G. Henssen

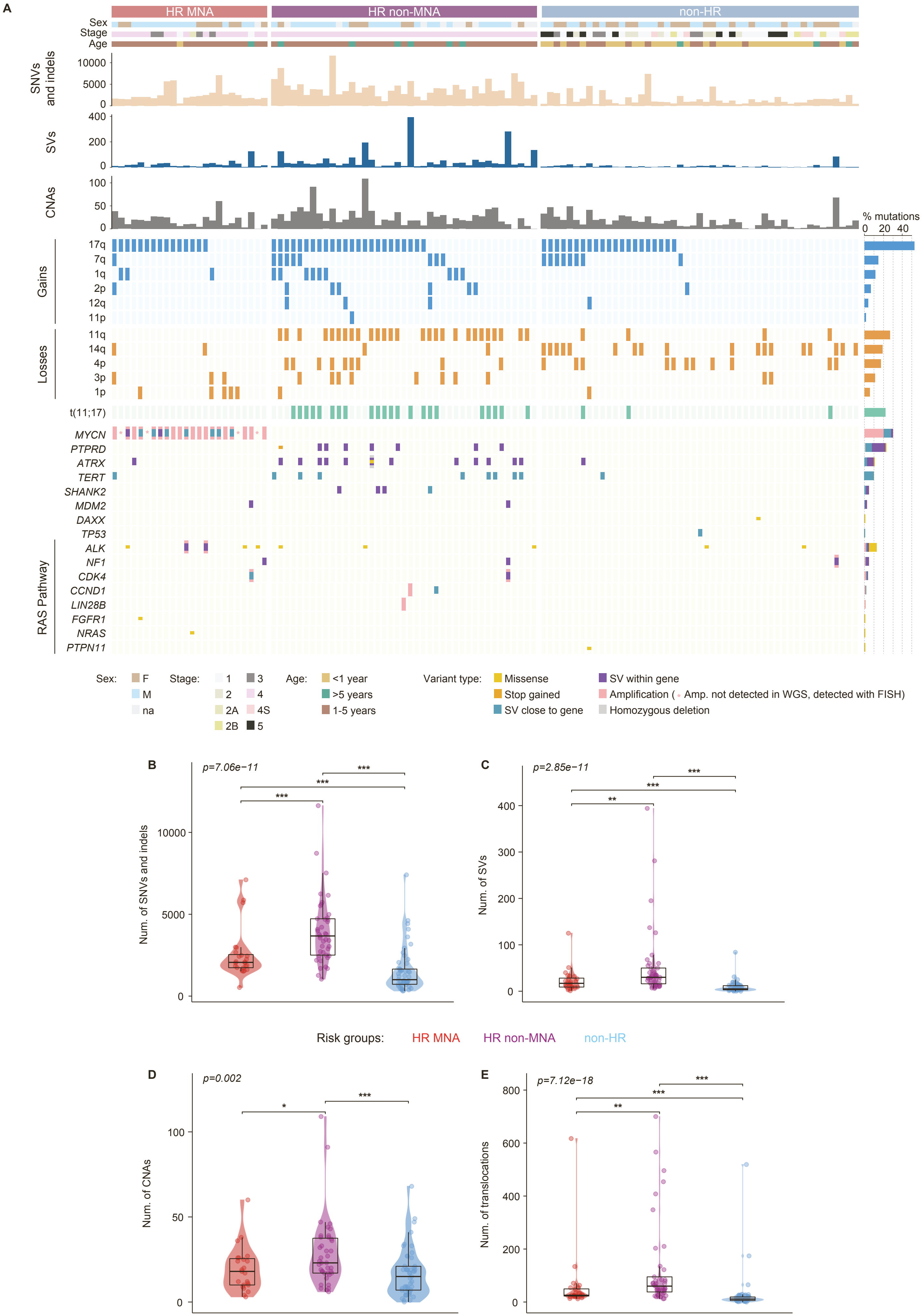
A
HR MNA
HR non-MNA
non-HR
Sex
Stage
Age
SNVs and indels
10000
5000
0
SVs
400
200
0
CNAs
100
50
0
% mutations
0 20 40
Gains
17q
7q
1q
2p
12q
11p
Losses
11q
14q
4p
3p
1p
t(11;17)
MYCN
PTPRD
ATRX
TERT
SHANK2
MDM2
DAXX
TP53
RAS Pathway
ALK
NF1
CDK4
CCND1
LIN28B
FGFR1
NRAS
PTPN11
Sex: F M na
Stage: 1 2 2A 2B 3 4 4S 5
Age: <1 year >5 years 1-5 years
Variant type: Missense Stop gained SV close to gene
SV within gene
Amplification (* Amp. not detected in WGS, detected with FISH)
Homozygous deletion
B
p=7.06e−11
***
***
***
Num. of SNVs and indels
10000
5000
0
C
p=2.85e−11
***
***
**
Num. of SVs
400
300
200
100
0
Risk groups: HR MNA HR non-MNA non-HR
D
p=0.002
*
***
Num. of CNAs
100
50
0
E
p=7.12e−18
***
***
**
Num. of translocations
800
600
400
200
0

Figure S1. Landscape of somatic variants in clinically heterogenous neuroblastomas, related to Figure 1.

(A) Top, risk group (HR MNA: high-risk *MYCN*-amplified, n = 24; HR non-MNA: high-risk non-*MYCN*-amplified, n = 41; non-HR: non-high-risk, n = 49), sex, stage of the disease, and age. Next, number of SNVs and indels, SVs, and CNAs in each of the 114 neuroblastoma patients from the discovery cohort. Middle, presence of known neuroblastoma chromosomal arm gains (blue), losses (orange), and translocations between chromosome 11 and 17 (green) in each patient. Bottom, presence of mutations in known neuroblastoma driver genes, including genes of the RAS pathway in each patient. Genes are ranked based on their mutation frequency in the cohort. The variant types considered here are SNVs and indels classified as missense and stop gained variants, SVs close and within the gene, amplifications, and homozygous deletions. Color indicates the type of mutation (key at the bottom). Patients with more than one type of variant in a gene have multiple colors designated. The right histogram indicates the percentage of mutations affecting each genomic feature in the whole cohort. Variant type is indicated by color. (B-E) Comparison between distributions of different variant types in the three neuroblastoma risk groups (HR MNA, HR non-MNA, non-HR) from the discovery cohort (n = 114). To assess if there are differences between risk groups, we used the non-parametric Kruskal-Wallis test (p-value in the upper left corner). The pairwise comparisons were done using the non-parametric Wilcoxon rank-sum test. (B) SNVs and indels, (C) SVs, (D) CNAs, and (E) translocations. Significance legend: *: p-value < 0.1, **: p-value < 0.05, ***: p-value < 0.01.

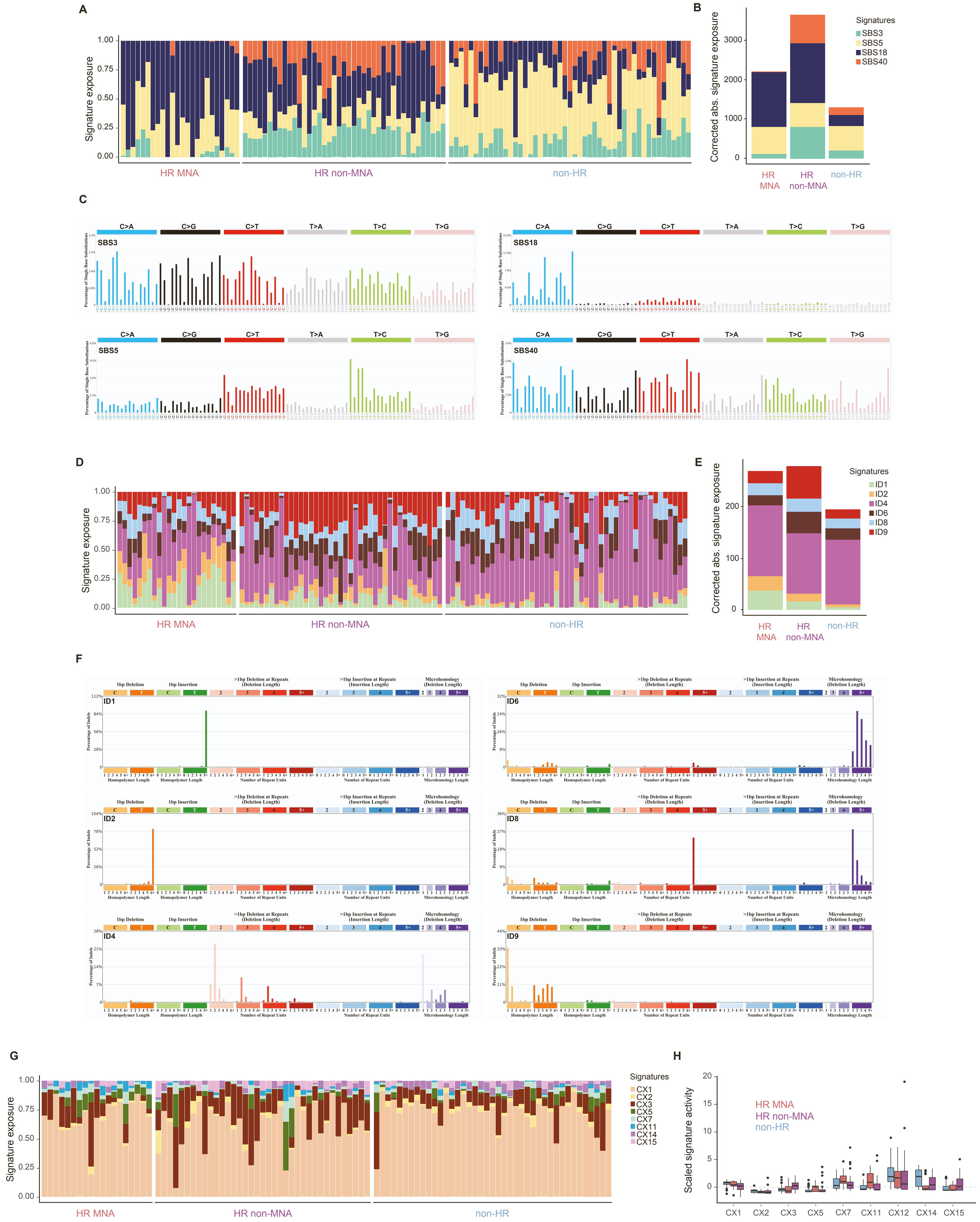
A
Signature exposure
1.00
0.75
0.50
0.25
0.00
HR MNA
HR non-MNA
non-HR
B
Corrected abs. signature exposure
3000
2000
1000
0
Signatures
SBS3
SBS5
SBS18
SBS40
HR MNA
HR non-MNA
non-HR
C
C>A
C>G
C>T
T>A
T>C
T>G
SBS3
SBS18
SBS5
SBS40
Percentage of Single Base Substitutions
D
Signature exposure
1.00
0.75
0.50
0.25
0.00
HR MNA
HR non-MNA
non-HR
E
Corrected abs. signature exposure
200
100
0
Signatures
ID1
ID2
ID4
ID6
ID8
ID9
HR MNA
HR non-MNA
non-HR
F
1bp Deletion
1bp Insertion
>1bp Deletion at Repeats (Deletion Length)
>1bp Insertion at Repeats (Insertion Length)
Microhomology (Deletion Length)
ID1
ID6
ID2
ID8
ID4
ID9
Percentage of Indels
Homopolymer Length
Number of Repeat Units
Microhomology Length
G
Signature exposure
1.00
0.75
0.50
0.25
0.00
Signatures
CX1
CX2
CX3
CX5
CX7
CX11
CX14
CX15
HR MNA
HR non-MNA
non-HR
H
Scaled signature activity
20
15
10
5
0
HR MNA
HR non-MNA
non-HR
CX1
CX2
CX3
CX5
CX7
CX11
CX12
CX14
CX15

b

Figure S2. SNV-, indel-, and CNA-based mutational signatures analysis, related to Figure 2. (A) Ratio of exposure of the six reference SNV-based signatures (SBS) identified in the discovery cohort for each of the 114 neuroblastoma patients. Patients have been grouped by risk (HR MNA, HR non-MNA, non-HR). Each color displays a different signature: SBS3, SBS5, SBS18, SBS40. (B) Absolute exposure of the four SNV-associated signatures (SBS) identified in the neuroblastoma discovery cohort by clinical risk group. Each color displays a different signature: SBS3, SBS5, SBS18, SBS40. (C) Mutational profiles for each of the reference SNV-based signatures detected in our cohort, reproduced from the COSMIC database (https://cancer.sanger.ac.uk/signatures/sbs/). (D) Ratio of exposure of the six reference indel-based signatures (ID) identified in the discovery cohort for each of the 114 neuroblastoma patients. Patients have been grouped by risk (HR MNA, HR non-MNA, non-HR). Each color displays a different signature: ID1, ID2, ID4, ID6, ID8, ID9. (E) Absolute exposure of the six indel-associated signatures (ID) identified in the neuroblastoma discovery cohort by clinical risk group. Each color displays a different signature: ID1, ID2, ID4, ID6, ID8, ID9. (F) Mutational profiles for each of the reference indel-based signatures detected in our cohort, reproduced from the COSMIC database (https://cancer.sanger.ac.uk/signatures/id/). (G) Ratio of exposure of the 8 CNA-associated signatures identified in the discovery cohort for each of the 114 neuroblastoma patients. Patients have been grouped by risk (HR MNA, HR non-MNA, non-HR). Each color displays a different signature: CX1, CX2, CX3, CX5, CX7, CX11, CX14, CX15. (H) Ratio of scaled signature activity the 8 CNA-associated signatures identified in our cohort for each of the 114 neuroblastoma patients. Scaling has been performed using PCAWG data.

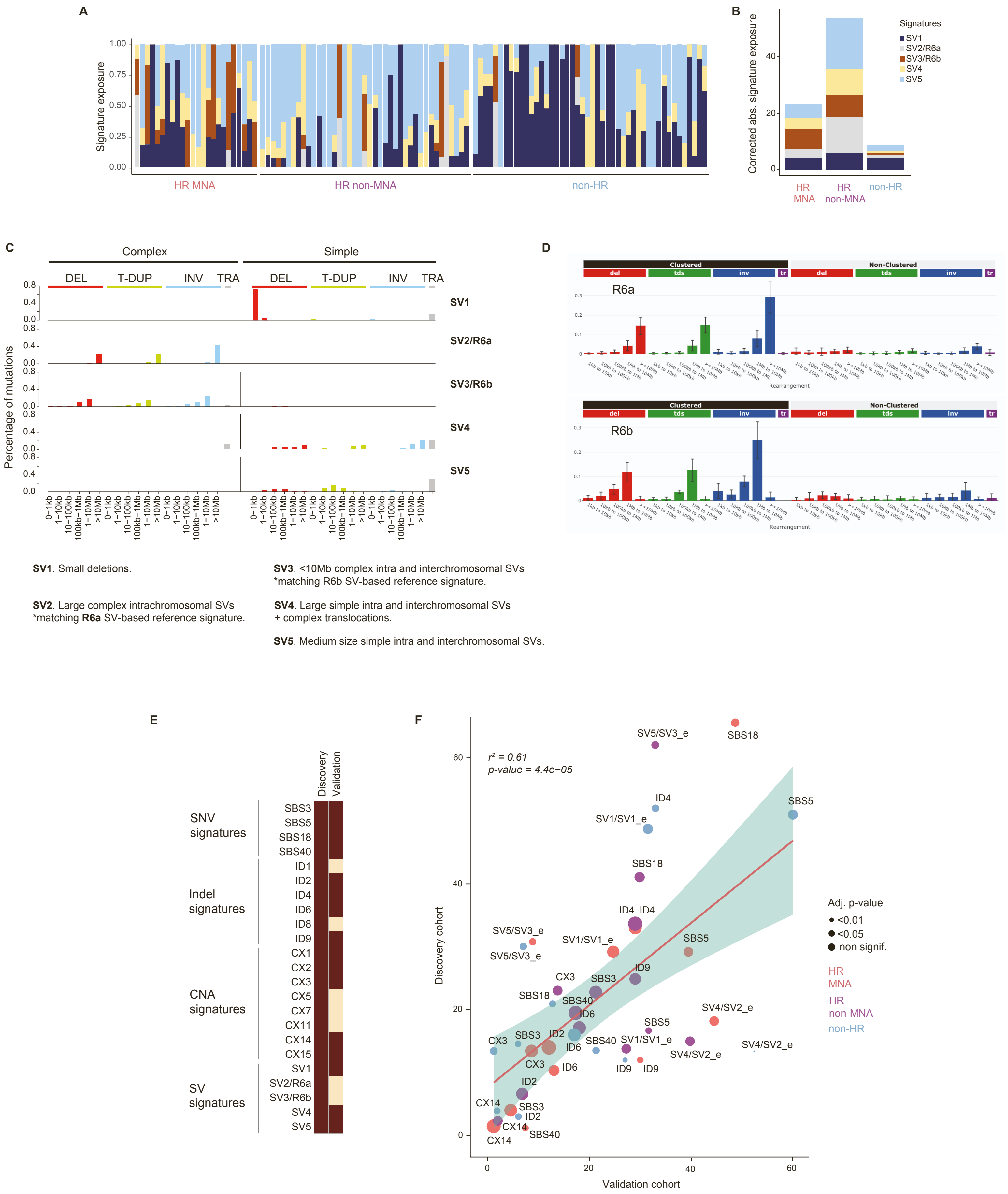
A
Signature exposure
1.00
0.75
0.50
0.25
0.00
HR MNA
HR non-MNA
non-HR
B
Corrected abs. signature exposure
0
20
40
HR MNA
HR non-MNA
non-HR
Signatures
SV1
SV2/R6a
SV3/R6b
SV4
SV5
C
Complex
Simple
DEL
T-DUP
INV
TRA
Percentage of mutations
0.0
0.4
0.8
SV1
SV2/R6a
SV3/R6b
SV4
SV5
0-1kb
1-10kb
10-100kb
100kb-1Mb
1-10Mb
>10Mb
D
Clustered
Non-Clustered
del
tds
inv
tr
R6a
R6b
Rearrangement
SV1. Small deletions.
SV2. Large complex intrachromosomal SVs
*matching R6a SV-based reference signature.
SV3. <10Mb complex intra and interchromosomal SVs
*matching R6b SV-based reference signature.
SV4. Large simple intra and interchromosomal SVs
+ complex translocations.
SV5. Medium size simple intra and interchromosomal SVs.
E
Discovery
Validation
SNV signatures
SBS3
SBS5
SBS18
SBS40
Indel signatures
ID1
ID2
ID4
ID6
ID8
ID9
CNA signatures
CX1
CX2
CX3
CX5
CX7
CX11
CX14
CX15
SV signatures
SV1
SV2/R6a
SV3/R6b
SV4
SV5
F
r² = 0.61
p-value = 4.4e−05
Discovery cohort
Validation cohort
0
20
40
60
Adj. p-value
<0.01
<0.05
non signif.
HR MNA
HR non-MNA
non-HR
SBS18
SV5/SV3_e
ID4
SBS5
SV1/SV1_e
SBS3
SBS40
ID2
ID6
ID9
CX3
CX14
SV4/SV2_e

Figure S3. SV-based mutational signatures analysis and comparison of mutational signatures from all the independent variant types in two different neuroblastoma cohorts, related to Figure 2.

(A) Ratio of exposure of the 5 SV-based signatures identified in the discovery cohort for each of the 114 neuroblastoma patients. Patients have been grouped by risk (HR MNA, HR non-MNA, non-HR). Each color displays a different signature: SV1-SV5. (B) Absolute exposure of the 5 SV-based signatures identified in the discovery cohort for each of the 114 neuroblastoma patients. Patients have been grouped by risk (HR MNA, HR non-MNA, non-HR). Each color displays a different signature: SV1-SV5. (C) Mutational profiles for each of the five SV-based signatures detected in our cohort, including a short description of their defining characteristics. (D) Mutational profiles for reference SV-based signatures R6a and R6b, corresponding to the *de novo* identified signatures SV2 and SV3, respectively. Reproduced from the Signal database (https://signal.mutationalsignatures.com/). (E) Plot depicting the detection status of the different reference mutational signatures in both the discovery (n = 114) and the validation (n = 36) cohorts. Dark red corresponds to presence, light yellow to absence. (F) Dot plot showing the linear correlation ($r^2 = 0.61$, $P = 4.4 \times 10^{-5}$) between the frequencies of exposures of the different mutational signatures in each risk group for each cohort. Each dot represents the correlation between the frequencies in both cohorts. Colors represent risk group classification. Size of the dot represents statistical differences in the frequencies between the cohorts (non-parametric Wilcoxon rank-sum test).

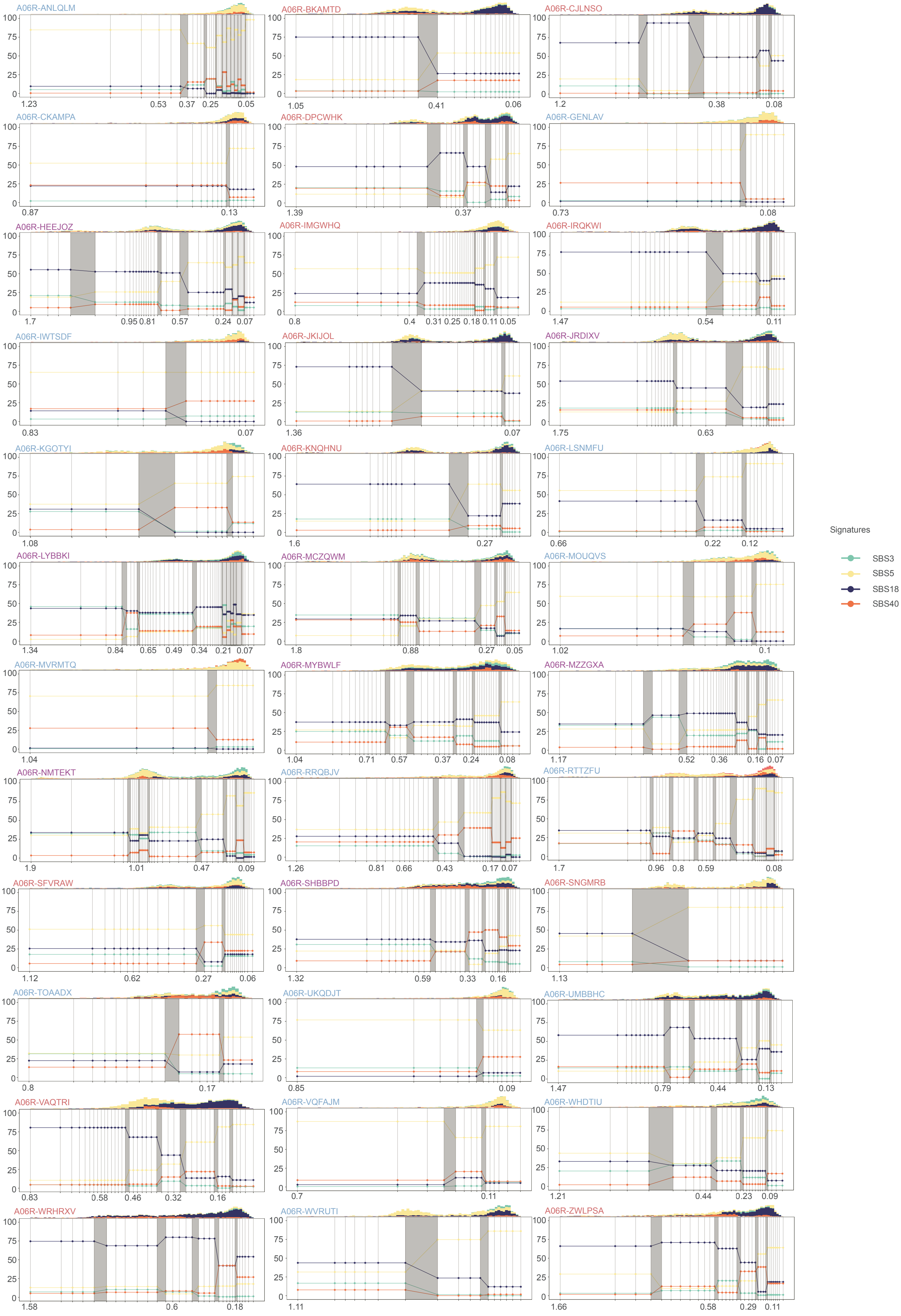

A06R-ANLQLM
A06R-BKAMTD
A06R-CJLNSO
A06R-CKAMPA
A06R-DPCWHK
A06R-GENLAV
A06R-HEEJOZ
A06R-IMGWHQ
A06R-IRQKWI
A06R-IWTSDF
A06R-JKIJOL
A06R-JRDIXV
A06R-KGOTYI
A06R-KNQHNU
A06R-LSNMFU
A06R-LYBBKI
A06R-MCZQWM
A06R-MOUQVS
A06R-MVRMTQ
A06R-MYBWLF
A06R-MZZGXA
A06R-NMTEKT
A06R-RRQBJV
A06R-RTTZFU
A06R-SFVRAW
A06R-SHBBPD
A06R-SNGMRB
A06R-TOAADX
A06R-UKQDJT
A06R-UMBBHC
A06R-VAQTRI
A06R-VQFAJM
A06R-WHDTIU
A06R-WRHRXV
A06R-WVRUTI
A06R-ZWLPSA
Signatures
SBS3
SBS5
SBS18
SBS40

Figure S4. Subclonal mutational signature trajectories by patient, related to Figure 2.

Activity trajectories of the four SNV-associated signatures (SBS) per cancer cell fraction by patient from the validation cohort (n = 36). For each plot, the y axis corresponds to the activity of mutational signatures (in %), and the x axis, the decreasing cancer cell fraction (CCF). Vertical grey lines indicate timepoints, the density of which increases with increasing mutation VAF density. Top histograms show different clusters of subclonal populations, with the frequency of each signature.

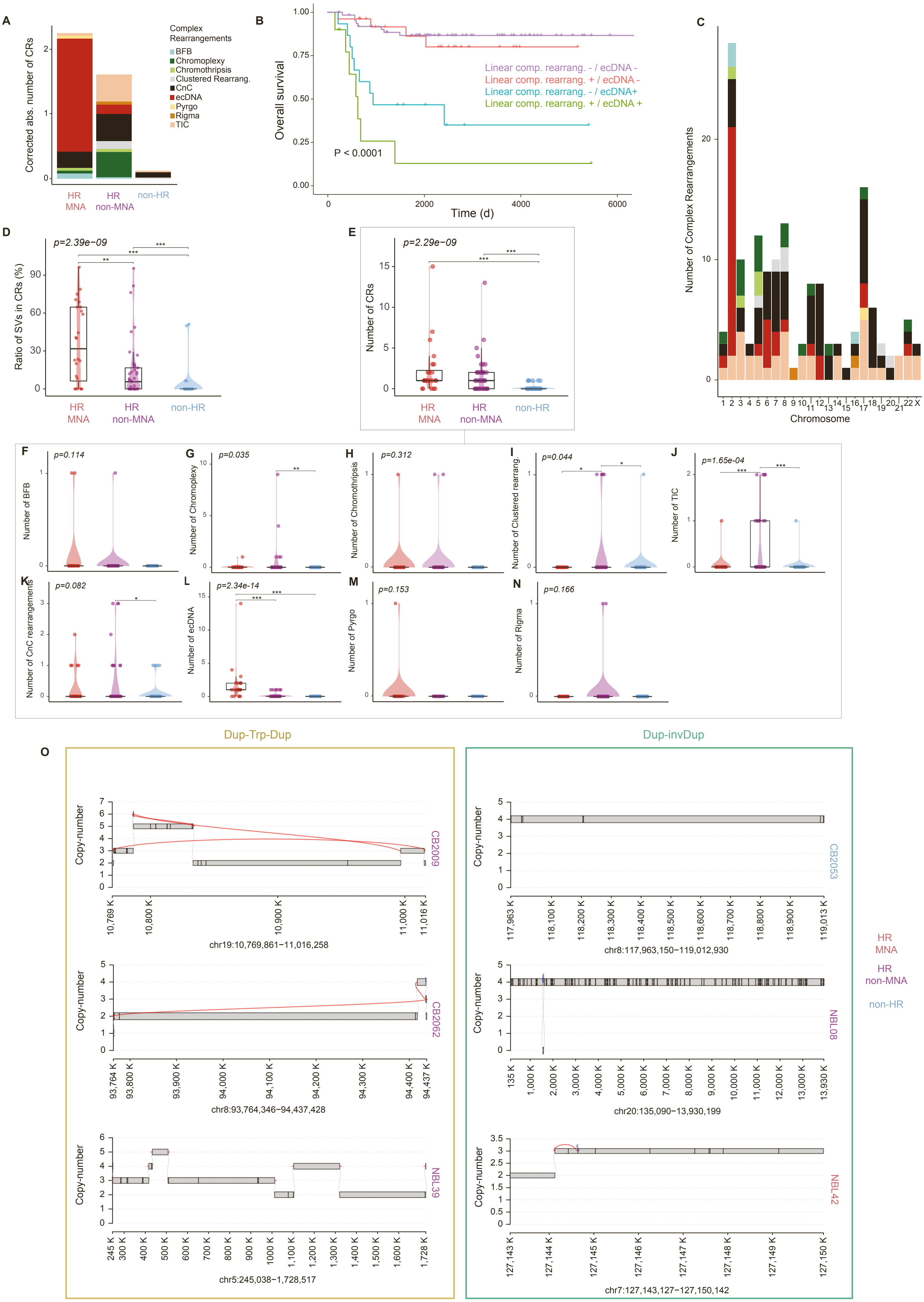

A
Corrected abs. number of CRs
Complex Rearrangements
BFB
Chromoplexy
Chromothripsis
Clustered Rearrang.
CnC
ecDNA
Pyrgo
Rigma
TIC
HR MNA
HR non-MNA
non-HR
B
Overall survival
Time (d)
Linear comp. rearrang. - / ecDNA -
Linear comp. rearrang. + / ecDNA -
Linear comp. rearrang. - / ecDNA+
Linear comp. rearrang. + / ecDNA +
P < 0.0001
C
Number of Complex Rearrangements
Chromosome
D
p=2.39e−09
Ratio of SVs in CRs (%)
E
p=2.29e−09
Number of CRs
F
p=0.114
Number of BFB
G
p=0.035
Number of Chromoplexy
H
p=0.312
Number of Chromothripsis
I
p=0.044
Number of Clustered rearrang.
J
p=1.65e-04
Number of TIC
K
p=0.082
Number of CnC rearrangements
L
p=2.34e-14
Number of ecDNA
M
p=0.153
Number of Pygo
N
p=0.166
Number of Rigma
O
Dup-Trp-Dup
Dup-invDup
Copy-number
CB2009
chr19:10,769,861−11,016,258
CB2062
chr8:93,764,346−94,437,428
NBL39
chr5:245,038−1,728,517
CB2053
chr8:117,963,150−119,012,930
NBL08
chr20:135,090−13,930,199
NBL42
chr7:127,143,127−127,150,142
HR MNA
HR non-MNA
non-HR

Figure S5. General characteristics of complex rearrangements, and reconstruction of the unclassified *clustered rearrangements* identified in the discovery cohort, related to Figures 4 and 5.

(A) Absolute count of the 9 complex rearrangement types identified in the discovery cohort for each of the 114 neuroblastoma patients. Patients have been grouped by risk (HR MNA, HR non-MNA, non-HR). Counts are corrected by the number of patients per risk group. Each color displays a complex rearrangement class. (B) Kaplan-Meier survival curve showing the overall survival of patients presenting ecDNA and linear complex rearrangements (green), only ecDNA (blue), only linear complex rearrangements (red) or no complex rearrangements (purple) ($P < 1 \times 10^{-4}$ by Log-rank test). (C) Distribution of the number of different complex rearrangement classes per chromosome. (D) Box plot comparing the ratio in percentage of structural variants (SVs) involved in complex rearrangements for each of the three neuroblastoma risk groups (HR MNA, HR non-MNA, non-HR). (E)-(N) Box plots comparing the number of complex rearrangements across the three neuroblastoma risk groups (HR MNA, HR non-MNA, non-HR). (E) for all complex rearrangement types, (F) BFB, (G) chromoplexy, (H) chromothripsis, (I) *clustered rearrangements*, (J) TIC, (K) complex non-cyclic, (L) ecDNA, (M) pyrgo, and (N) rigma. Each dot represents a patient. To assess if there are differences between risk groups, we used the non-parametric Kruskal-Wallis test (p-value in the upper left corner). The pairwise comparisons were done using the non-parametric Wilcoxon rank-sum test. Significance legend: *: p-value < 0.1, **: p-value < 0.05, ***: p-value < 0.01. (O) Reconstruction of the *clustered rearrangements* detected in the neuroblastoma discovery cohort , based on the copy number of the segments and the junctions connecting these segments. Each plot corresponds to a different patient. The plots are classified in two columns by the complex SV type: duplication-inverted-triplication-duplication (Dup-Trp-Dup) and duplications linked by inverted segments (Dup-invDup).
